# AMPAR Subunit Gene Expression Marks a Synaptic Transcriptional State in Lower-Grade Glioma

**DOI:** 10.3390/brainsci16080773

**Published:** 2026-07-23

**Authors:** Bruno Rodrigues, Matheus Dalmolin, Henrique Ritter Dal-Pizzol, Osvaldo Malafaia, Marcelo A. C. Fernandes, Karina Munhoz de Paula Alves Coelho, Rafael Roesler, Gustavo R. Isolan

**Affiliations:** 1 Graduate Program in Principles of Surgery, Mackenzie Evangelical University, Curitiba 80730-000, Brazil; 2 InovAI Lab, nPITI/IMD, Federal University of Rio Grande do Norte, Natal 59078-970, Brazil; 3 Bioinformatics Multidisciplinary Environment (BioME), Federal University of Rio Grande do Norte, Natal 59078-970, Brazil; 4 National Science and Technology Institute for Children’s Cancer Biology and Pediatric Oncology—INCT BioOncoPed, Porto Alegre 90035-003, Brazil; 5 Cancer and Neurobiology Laboratory, Experimental Research Center, Clinical Hospital (CPE-HCPA), Federal University of Rio Grande do Sul, Porto Alegre 90035-903, Brazil; 6 Department of Computer Engineering and Automation, Federal University of Rio Grande do Norte, Natal 59078-970, Brazil; 7 Department of Scientific Development and Innovation (DECIPE), Center for Anatomo-Pathological Diagnosis (CEDAP), Joinville 89200-000, Brazil; 8 Graduate Program in Medicine: Medical Sciences, Faculty of Medicine, Federal University of Rio Grande do Sul, Porto Alegre 90035-003, Brazil; 9 Department of Pharmacology, Institute for Basic Health Sciences, Federal University of Rio Grande do Sul, Porto Alegre 90035-003, Brazil; 10 Center for Biotechnology, Federal University of Rio Grande do Sul, Porto Alegre 91501-970, Brazil; 11 The Center for Advanced Neurology and Neurosurgery (CEANNE), Porto Alegre 90560-010, Brazil

**Keywords:** AMPA receptor, glutamate receptor, *GRIA* gene, lower-grade glioma, brain tumor, cancer neuroscience

## Abstract

**Highlights:**

**What are the main findings?**

- Expression of the genes that encode AMPA receptor (AMPAR) subunits, *GRIA1*–*GRIA4*, is correlated with a broad synaptic gene network in lower-grade glioma (LGG).
- High *GRIA1*–*GRIA4* expression is associated with favorable clinicomolecular features and longer overall survival (OS) in LGG patients.

**What are the implications of the main findings?**

- AMPAR subunit gene expression is associated with a neuronal/synaptic transcriptional state in LGG.
- These findings support further investigation of neuron–glioma interactions and synaptic signaling pathways as determinants of glioma biology.

**Abstract:**

**Background:** Glutamatergic neuron-to-glioma signaling mediated by α-amino-3-hydroxy-5-methyl-4-isoxazolepropionic acid receptors (AMPARs) has emerged as an important mechanism in glioma progression. **Objectives/Methods:** We analyzed the expression of the AMPAR subunit genes *GRIA1*, *GRIA2*, *GRIA3*, and *GRIA4* in lower-grade glioma (LGG). **Results:** Expression of *GRIA1*–*GRIA4* was highest in IDH-mutant/1p19q-codeleted tumors and lowest in IDH-wildtype tumors across both The Cancer Genome Atlas (TCGA) and the Chinese Glioma Genome Atlas (CGGA) cohorts. High expression of each *GRIA* gene was associated with longer overall survival (OS). Transcriptome-wide analyses identified positive correlations between an AMPAR score and genes involved in synaptic organization, neuronal connectivity, and neurotransmission. Co-expression analyses demonstrated coordinated expression between *GRIA1*–*GRIA4* and genes encoding AMPAR auxiliary proteins. Gene Ontology (GO) enrichment revealed overrepresentation of synaptic signaling, trans-synaptic communication, and synapse organization. Although the AMPAR score was associated with favorable survival in univariate analyses, it did not retain independent prognostic significance after adjustment for key clinicomolecular variables. Elevated expression of AMPAR subunit genes in LGG was associated with favorable molecular subtypes and a synaptic transcriptional program. **Conclusions:** These findings suggest that *GRIA1*–*GRIA4* expression is associated with a synaptically enriched transcriptional program in LGG, although its cellular origin remains uncertain.

## 1. Introduction

Increasing attention has been given to the structural and functional interactions between brain tumors and surrounding neurons. Glioma initiation, progression, and invasion are strongly influenced by neural activity, the formation of neuron–glioma synapses, and the integration of glioma cells into neural circuits [1,2,3,4,5,6]. Glutamatergic signaling mediated by α-amino-3-hydroxy-5-methyl-4-isoxazolepropionic acid (AMPA) ionotropic receptors (AMPARs) represents a key mechanism underlying neuron-to-glioma synaptic communication [7,8,9,10,11].

AMPARs are composed of four distinct subunits that assemble into a tetrameric structure, forming a sodium-permeable ligand-gated ion channel responsible for most fast excitatory neurotransmission in the central nervous system (CNS) [12]. Receptor subunits are encoded by four genes located in different chromosomes, namely *GRIA1* (which encodes GluA1 or GluR1), *GRIA2* (GluA2 or GluR2), *GRIA3* (GluA3 or GluR3), and *GRIA4* (GluA4 or GluR4) [13] (Table 1). GluR2 is the main subunit determining AMPAR biophysical properties, including calcium permeability and channel conductance, whereas GluR1 strongly influences synaptic plasticity underlain by long-term potentiation (LTP), and GluR4 is more prominent during early CNS development [14,15]. In the hippocampus, a brain area crucially involved in neural plasticity mediated by glutamatergic transmission, the most abundant AMPAR subtypes are GluR1/2 and GluR2/3 heterotetramers, with GluR1/2 heteromers being the dominant AMPAR subtype at hippocampal CA1 cell synapses. Each receptor subtype contributes differentially to synaptic plasticity, due to influences from specific associated proteins [16,17,18,19].

Glioma cells express all four AMPAR subunits, and glutamatergic neuron-to-glioma synapses in glioma grade IV (glioblastoma, GBM), which is the most aggressive primary brain tumor in adults, are mediated by AMPAR activity [4]. Pharmacological blockade of AMPARs by compounds including the selective antagonist perampanel affects the growth and invasion of experimental GBM [9,10,20,21]. Perampanel has also been recently tested in the clinical setting in a pilot trial based on the glutamatergic neuron–glioma synaptogenesis to modulate peritumoral hyperexcitability [22]. Another AMPAR antagonist, talampanel, has been evaluated in a phase 2 trial in patients with recurrent GBM or anaplastic gliomas, with the results indicating that the drug was well-tolerated but had no significant activity when given as a single agent [23].

Despite these pharmacological investigations, relatively few studies have examined the influence of gene or protein expression of AMPAR subunits on glioma. In a previously studied tissue array, GluR1 was significantly more expressed in GBM compared to anaplastic astrocytoma and low-grade tumors. In addition, reduction in GluR1 protein expression in GBM cells inhibited cell proliferation [24]. Venkatesh et al. [6] reported broad *GRIA* gene expression in tumors from patients with different subtypes of GBM and enrichment of gene expression within distinct malignant cell subpopulations. However, the possible association of *GRIA* genes with patient prognosis remains poorly understood. Moreover, almost all previous studies have focused on GBM, whereas the role of AMPARs in lower grade gliomas (LGGs) remains unknown. LGGs are generally less aggressive brain tumor types compared to primary GBM that occurs at an earlier age in adulthood; however, they undergo increasing transformation, giving rise to highly malignant gliomas over time [25,26,27]. Here, we investigated associations between *GRIA* gene expression, patient overall survival (OS), and a synaptic transcriptional signature in LGG.

## 2. Materials and Methods

### 2.1. Datasets and Gene Expression Analyses

RNA-sequencing data and associated clinical information from patients with LGG were collected from two independent public cohorts comprising World Health Organization (WHO) grade 2 and grade 3 diffuse gliomas: The Cancer Genome Atlas (TCGA-LGG; https://www.cancer.gov/tcga) [28] and the Chinese Glioma Genome Atlas (CGGA; http://www.cgga.org.cn) [29]. For the TCGA cohort, gene-level expression counts were obtained from the TOIL recompute project through the UCSCXenaTools package, while clinical and molecular annotations were retrieved from the TCGA Pan-Cancer Atlas using the cBioPortalData package. For the CGGA cohort, RNA-sequencing and clinical datasets from the CGGA-325 and CGGA-693 projects were downloaded and integrated. Only genes present in both datasets were retained for downstream analyses. To minimize the impact of technical differences between the two CGGA datasets, dataset origin (CGGA-325 versus CGGA-693) was included as a covariate in DESeq2-based analyses. Cases lacking essential clinical information or survival data were excluded.

TCGA expression data were converted from expected counts to raw count estimates to enable compatibility with count-based analytical approaches implemented in DESeq2. For the CGGA cohort, count matrices from both studies were combined after harmonization of gene identifiers. When duplicate gene symbols were encountered, the transcript with the highest average expression across samples was retained. Normalized count data were subjected to variance-stabilizing transformation using DESeq2, followed by gene-wise standardization through z-score transformation within each cohort.

Expression patterns of the AMPAR subunit genes *GRIA1*, *GRIA2*, *GRIA3*, and *GRIA4* were investigated across molecularly defined LGG subgroups according to the current integrated classification framework [30,31]. Tumors were categorized as IDH-mutant with 1p/19q codeletion (LGG-IDH-mut-codel, corresponding to oligodendroglioma; TCGA, *n* = 164; CGGA, *n* = 113), IDH-mutant without 1p/19q codeletion (LGG-IDH-mut-non-codel, corresponding to astrocytoma; TCGA, *n* = 235; CGGA, *n* = 142), or IDH-wildtype (LGG-IDH-wt; TCGA, *n* = 91; CGGA, *n* = 88). Differences in gene expression among groups were assessed using Wilcoxon rank-sum tests for pairwise comparisons, and multiple-testing correction was performed using the False Discovery Rate (FDR) Benjamini–Hochberg (BH) procedure.

### 2.2. Gene Expression Correlation and Functional Enrichment Analyses

RNA-sequencing data for LGG tumors were obtained from TCGA through the TCGAbiolinks package in R. Gene-level expression values were extracted from the FPKM-UQ normalized expression matrix (fpkm_uq_unstrand). Genes were annotated using HGNC gene symbols provided in the TCGA annotation files. When multiple Ensembl identifiers mapped to the same gene symbol, duplicate entries were removed.

The core AMPAR gene set consisted of *GRIA1*, *GRIA2*, *GRIA3*, and *GRIA4*. Genes encoding AMPAR auxiliary subunits and interacting proteins included *CACNG2*, *CACNG3*, *CACNG4*, *CACNG5*, *CACNG7*, *CACNG8*, *CNIH2*, *CNIH3*, *GSG1L*, *SHISA6*, *SHISA7*, and *SHISA9*. Expression values were log_2_-transformed prior to analysis. Pairwise gene–gene co-expression was assessed using Spearman rank correlation coefficients across all TCGA-LGG samples. For candidate-gene analyses, correlations were calculated between each GRIA subunit gene and each AMPAR auxiliary protein.

To capture the overall transcriptional activity of genes encoding receptor subunits, a composite AMPAR expression score (AMPAR score) was calculated for each tumor as the arithmetic mean of the normalized log_2_-transformed expression values of *GRIA1*, *GRIA2*, *GRIA3*, and *GRIA4*. This composite metric allowed to represent overall AMPAR subunit gene expression while reducing the influence of variability in individual subunits. The AMPAR score was subsequently used for transcriptome-wide correlation analyses, functional enrichment analyses, and multivariable survival modeling. For transcriptome-wide analyses, Spearman correlation coefficients were calculated between the AMPAR expression score and expression levels of all genes in the transcriptome. Genes demonstrating positive correlations with the AMPAR score were ranked according to correlation strength and subjected to Gene Ontology (GO) enrichment analysis using g:Profiler via the gprofiler2 package. Biological Process and Cellular Component ontologies were evaluated. Statistical significance was determined using the multiple-testing correction implemented by g:Profiler.

To determine whether the observed co-expression program was primarily attributable to differences in glioma molecular subtype composition, additional analyses were performed using two complementary approaches. First, transcriptome-wide co-expression analysis was repeated after statistically adjusting gene expression values for molecular subtype, and the resulting subtype-adjusted correlations were visualized as a clustered heatmap. Second, transcriptome-wide co-expression analyses were performed independently within each major TCGA-LGG molecular subtype (IDH-mutant/1p19q-codeleted, IDH-mutant/non-codeleted, and IDH-wildtype) as well as within WHO grade II and grade III tumors (Supplementary Figure S3). For each subgroup, a subgroup-specific AMPAR expression score was calculated as the mean expression of *GRIA1*, *GRIA2*, *GRIA3*, and *GRIA4*, followed by transcriptome-wide Spearman correlation analysis. The 30 genes showing the strongest positive correlation with the subgroup-specific AMPAR score were identified, and their correlations with the individual AMPAR subunit genes were displayed as clustered heatmaps using the same analytical workflow applied to the overall TCGA-LGG cohort.

### 2.3. Survival Analysis

Associations between overall survival (OS) of patients with LGG and expression analyses of *GRIA1*–*GRIA4* were assessed using Kaplan–Meier survival curves. Patients were dichotomized into “high” and “low” expression groups based on the median. Survival distributions were compared using the log-rank test, with *p*-values adjusted for multiple testing across all genes and cohorts using the FDR BH method.

All analyses were performed in the R statistical environment (version 4.5.1). Variance-stabilizing transformation and batch adjustment were conducted using DESeq2 (version 1.48.1). OS analyses were performed using the survival (version 3.8.3), with expression cutpoints determined by the median. Kaplan–Meier curves were generated using survminer. Graphical visualizations were produced using ggplot2 (version 4.0.2). Data acquisition was performed using UCSCXenaTools (version 1.7.0) and cBioPortalData (version 2.20.0).

### 2.4. Multivariate Cox Analysis

Multivariable survival analysis was performed using Cox proportional hazards regression models. An AMPAR score was calculated for each tumor as the mean log_2_-transformed expression of *GRIA1*, *GRIA2*, *GRIA3*, and *GRIA4*. Overall survival was modeled as a function of the AMPAR score, age at diagnosis, histological grade, IDH status, and 1p/19q codeletion status. Hazard ratios (HRs) and 95% confidence intervals (CIs) were estimated using the Cox proportional hazards model.

To investigate the relationship between the AMPAR subunit gene transcriptional program and established molecular subtypes of LGG, AMPAR scores were compared among IDH-mutant/1p19q-codeleted, IDH-mutant/non-codeleted, and IDH-wildtype tumors. Group differences were assessed using the Kruskal–Wallis test followed by pairwise Wilcoxon rank-sum tests with FDR BH correction for multiple comparisons.

### 2.5. Statistical Analysis

Statistically significant differences were defined by BH FDR *p* values of less than 0.05. Visualization of the data, including violin plots overlaid with box plots and statistical summaries, were generated using the ggstatsplot and ggplot2 R packages. Survival curves were generated using the survival and survminer R packages.

### 2.6. Tumor Cellular Composition and Cell-Type Specificity of the GRIA Gene Signature

To determine whether the AMPAR subunit transcriptional signature could be explained by differences in tumor cellular composition, complementary bulk transcriptomic deconvolution and single-cell analyses were performed. The composite AMPAR score was calculated as the mean of the standardized (z-score transformed) expression values of *GRIA1*, *GRIA2*, *GRIA3*, and *GRIA4* for each glioma sample.

### 2.7. Stromal and Immune Infiltration Analysis

Stromal and immune infiltration were estimated using the ESTIMATE algorithm implemented in the R package estimate (version 1.0.13). The TCGA-LGG expression matrix was reformatted according to package requirements, and stromal, immune, and ESTIMATE scores were calculated using the estimateScore function. Associations between these scores and the AMPAR score were evaluated using Spearman’s rank correlation coefficient.

### 2.8. Cell-Type Deconvolution

Cell-type enrichment analysis was performed using the xCell algorithm implemented in the R package xCell (https://comphealth.ucsf.edu/app/xcell), which estimates the relative enrichment of 67 immune and stromal cell populations from bulk transcriptomic data. xCell analysis was performed in RNA-seq mode using the TCGA-LGG expression matrix. Spearman’s rank correlation coefficients were calculated between *GRIA1*, *GRIA2*, *GRIA3*, and *GRIA4* and each xCell cell-type enrichment score. Multiple comparisons were corrected using BH FDR.

### 2.9. Single-Cell RNA-Sequencing Analysis

To determine whether AMPAR subunit genes are expressed by malignant glioma cells, expression of *GRIA1*, *GRIA2*, *GRIA3*, and *GRIA4* was examined in a publicly available single-cell RNA-sequencing dataset of IDH-mutant astrocytoma (SCP50; Broad Institute Single Cell Portal; https://singlecell.broadinstitute.org/single_cell/study/SCP50/single-cell-rna-seq-analysis-of-astrocytoma). Expression patterns were evaluated across the annotated cellular populations provided by the dataset, including malignant cells, oligodendrocytes, microglia/macrophages, and T cells, using the portal’s interactive visualization tools, including t-SNE expression maps and violin plots.

### 2.10. Software, Artificial Intelligence, and Code Availability

Statistical analyses were performed in the R statistical environment (version 4.5.1). The principal packages used included DESeq2 (version 1.48.1), survival (version 3.8.3), survminer, ggplot2 (version 4.0.2), UCSCXenaTools (version 1.7.0), cBioPortalData (version 2.20.0), estimate (version 1.0.13), xCell, gprofiler2, and ggstatsplot. Analyses using publicly available datasets from TCGA, CGGA, and the Broad Institute Single Cell Portal were performed using the methods described in this manuscript. No custom software was developed for this study. The analyses were performed using publicly available R packages and publicly available datasets as described in the Methods.

During preparation of the manuscript, OpenAI’s ChatGPT (GPT-5.5) was used to assist in the graphical design of Figure 12. The underlying data analyses, scientific interpretation, figure content, and final editing were performed and verified by the authors, who take full responsibility for the published work.

## 3. Results

### 3.1. GRIA1–GRIA4 Expression Across LGG Subtypes

**Figure 1:**
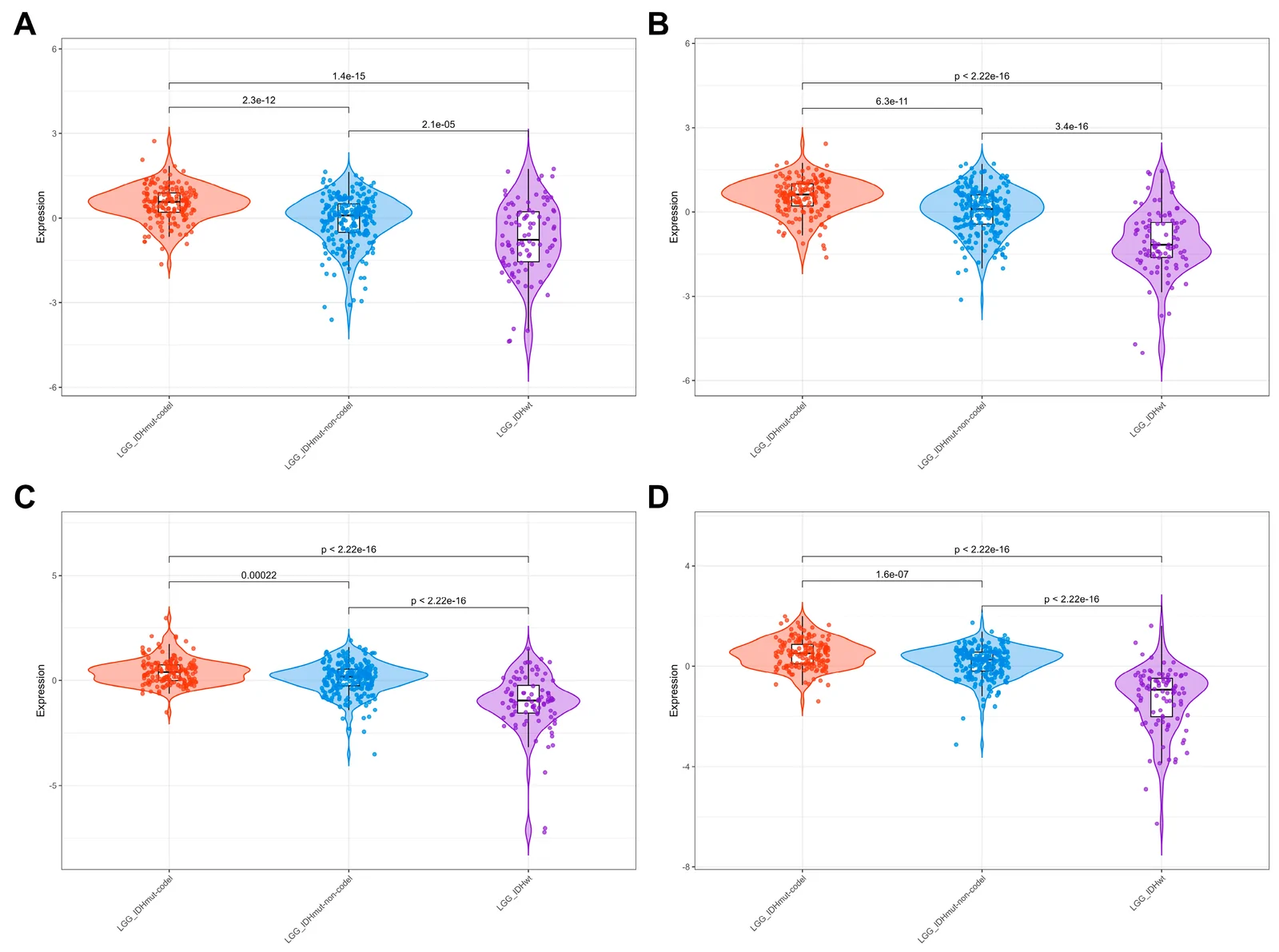
Gene expression levels of (**A**), *GRIA1*, (**B**), *GRIA2*, (**C**), *GRIA3*, and (**D**), *GRIA4* in TCGA LGG tumors classified into molecular subtypes. LGG-IDH-mut-codel, *n* = 164; LGG-IDH-mut-non-codel, *n* = 235; LGG-IDH-wt, *n* = 91. BH FDR *p*-values are indicated in the panels.

**Figure 2:**
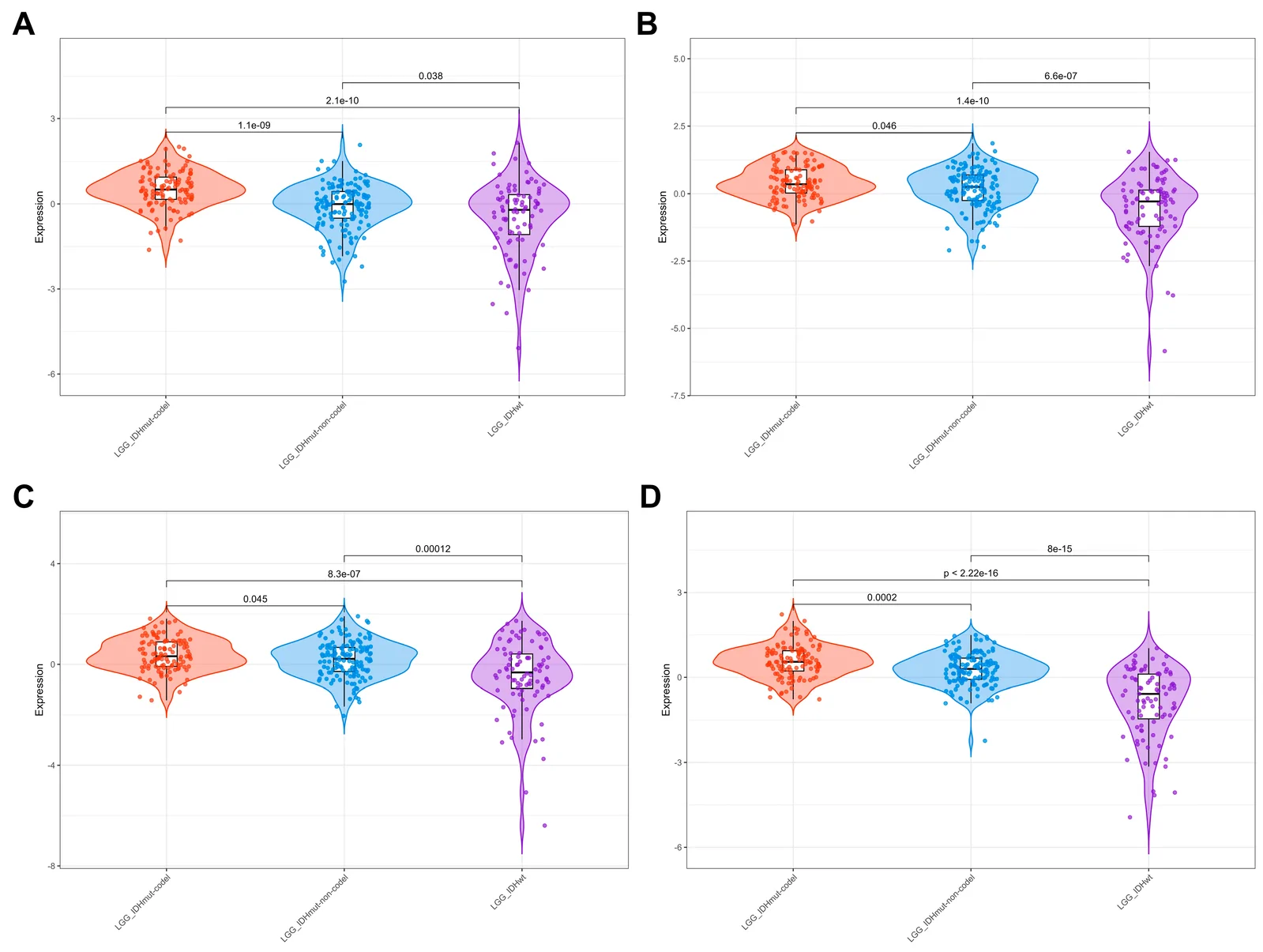
Gene expression levels of (**A**), *GRIA1*, (**B**), *GRIA2*, (**C**), *GRIA3*, and (**D**), *GRIA4* in CGGA tumors classified into molecular subtypes. LGG-IDH-mut-codel, *n* = 113; LGG-IDH-mut-non-codel, *n* = 142; LGG-IDH-wt, *n* = 88. BH FDR *p*-values are indicated in the panels.

We first analyzed the levels of *GRIA1*–*GRIA4* expression in TCGA LGG and CGGA tumors classified into molecular subtypes. For all four *GRIA* genes in both datasets, we found the highest expression in LGG-IDH-mut-codel and the lowest expression levels in LGG-IDH-wt, with intermediate levels in LGG-IDH-mut-non-codel (Figure 1 and Figure 2).

### 3.2. Transcriptome-Wide Analysis Reveals Strong Associations Between AMPAR Subunits and Other Synaptic Genes

**Figure 3:**
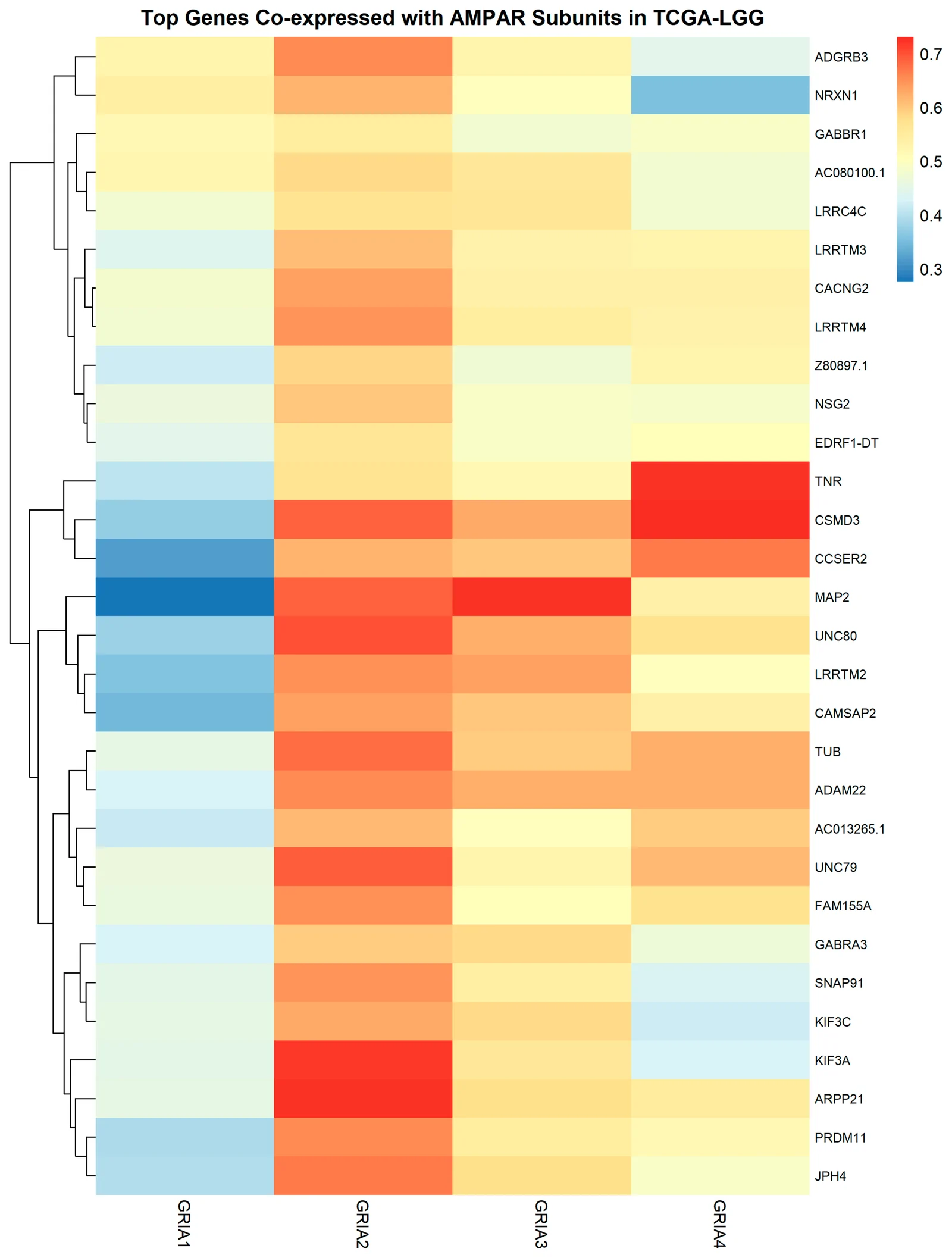
Top genes associated with the AMPAR transcriptional program in TCGA-LGG. Heatmap showing the strongest transcriptome-wide correlates of the AMPAR expression score. Genes are clustered according to similarity of correlation patterns across *GRIA1*–*GRIA4*.

Unbiased transcriptome-wide correlation analysis identified a set of genes strongly associated with *GRIA1*–*GRIA4* expression. Highest-ranking correlates included *CACNG2*, *ADAM22*, *LRRTM2*, *LRRTM3*, *LRRTM4*, *NRXN1*, *SNAP91*, and *MAP2*, indicating a pattern of genes encoding proteins involved predominantly in synaptic organization, neurotransmission, and neuronal connectivity (Figure 3).

To investigate whether the neuronal transcriptional program associated with AMPAR expression primarily reflected differences in glioma molecular subtype composition, we first examined the stability of this transcriptional program by repeating the transcriptome-wide co-expression analyses independently within each major TCGA-LGG molecular subtype (LGG IDH-mut-codel, LGG IDH-mut-non-code, and LGG IDH-wt), as well as within WHO grade II and grade III tumors. Although the identity of the genes showing the strongest positive correlations with the AMPAR score differed among subtypes, each analysis consistently identified genes involved in neuronal differentiation, synaptic organization, and neurotransmission (Supplementary Figures S1 and S3). Several neuronal and synaptic genes were recurrently identified across multiple subgroup analyses, including *ADAM22*, *TNR*, *CSMD3*, *LRRTM2*, *LRRTM3*, *LRRTM4*, *ADGRB3*, *NRXN1*, *UNC79*, *UNC80*, *MAP2*, *ARPP21*, *CAMSAP2*, *CACNG2*, and *KIF3A*, although their relative ranking varied according to molecular subtype and tumor grade. The LGG IDH-mut-non-codel tumors displayed a co-expression profile most similar to that observed in the overall TCGA-LGG cohort, whereas IDH-wt tumors retained a neuronal transcriptional signature but exhibited enrichment of a partially distinct set of neuronal genes, including *ELFN2*, *GLRB*, *NTRK3*, *CACNA1D*, and *CACNA2D2*. Likewise, grade II and grade III tumors shared a common neuronal/synaptic transcriptional framework while differing in the specific genes most strongly associated with AMPAR expression.

We also repeated the transcriptome-wide co-expression analysis after statistically adjusting gene expression values for molecular subtype. The resulting subtype-adjusted heatmap demonstrated that the characteristic neuronal/synaptic co-expression pattern remained largely preserved following removal of subtype-associated variation (Supplementary Figure S2). Several of the genes most strongly associated with the AMPAR score in the overall cohort, including *UNC80*, *MAP2*, *LRRTM2*, *TNR*, *CSMD3*, *UNC79*, *CAMSAP2*, *LRRTM3*, *ADAM22*, *PRDM11*, *KIF3A*, *LRRTM4*, *LRRC4C*, *KIF3C*, *NRXN1*, *ADGRB3*, *ARPP21*, *GABRA3*, *SNAP91*, *CACNG2*, *GABBR1*, and *NSG2*, continued to show coordinated correlations with the AMPAR subunit genes after subtype adjustment, indicating that differences in tumor molecular subtype composition do not fully account for the observed transcriptional program.

Together, these complementary analyses demonstrate that the AMPAR-associated neuronal transcriptional program is overall preserved following statistical adjustment for molecular subtype and is also maintained within individual molecular subtypes and histological grades. While the composition of the co-expression network varies according to tumor context, its predominant neuronal and synaptic functional identity remains consistently detectable across clinically relevant LGG subgroups.

### 3.3. The AMPAR Subunit Gene-Centered Network Contains an Interconnected Module of Synaptic Genes

**Figure 4:**
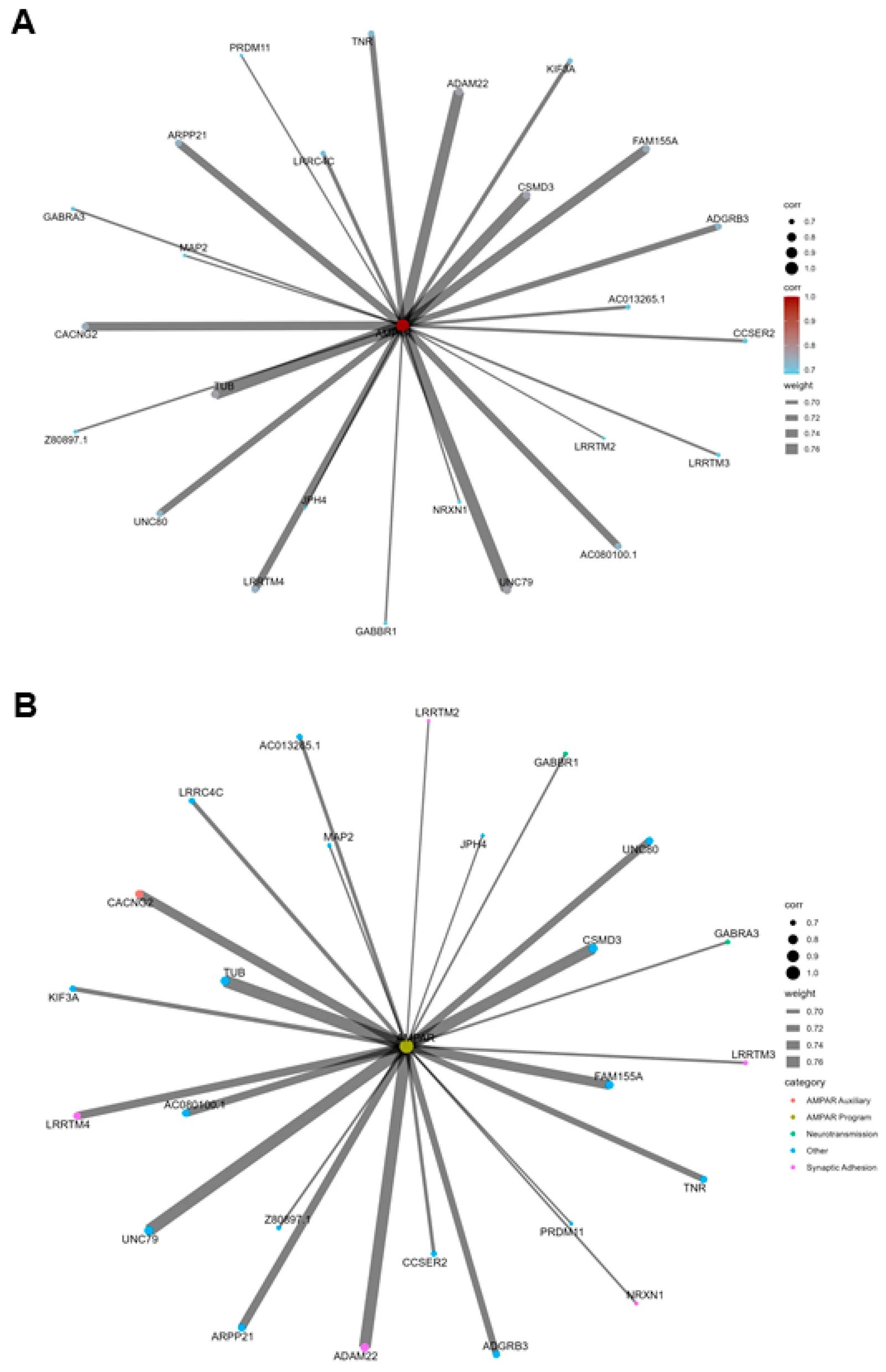
Network representation of the AMPAR-associated transcriptional program in TCGA LGG tumors. The graphs depict the strongest transcriptome-wide correlates of the AMPAR expression score. (**A**), node size is proportional to correlation strength, and edges connect genes to the AMPAR program. Prominent nodes include *CACNG2*, *ADAM22*, *LRRTM2*, *LRRTM3*, *LRRTM4*, and *NRXN1*, emphasizing enrichment for synaptic organization pathways. (**B**), colors identify AMPAR auxiliary proteins and signaling, neurotransmission, and synaptic adhesion as the main biological categories.

Network visualization of the strongest AMPAR subunit-associated genes revealed a highly interconnected module centered on synaptic signaling components. Key nodes included *CACNG2*, *ADAM22*, *LRRTM* family members, and *NRXN1*, and highlighted transcriptional coupling between AMPAR subunit expression and genes involved in synaptic adhesion and organization (Figure 4).

### 3.4. AMPAR Subunit Genes Show Coordinated Expression with Genes Encoding AMPAR Auxiliary Proteins

**Figure 5:**
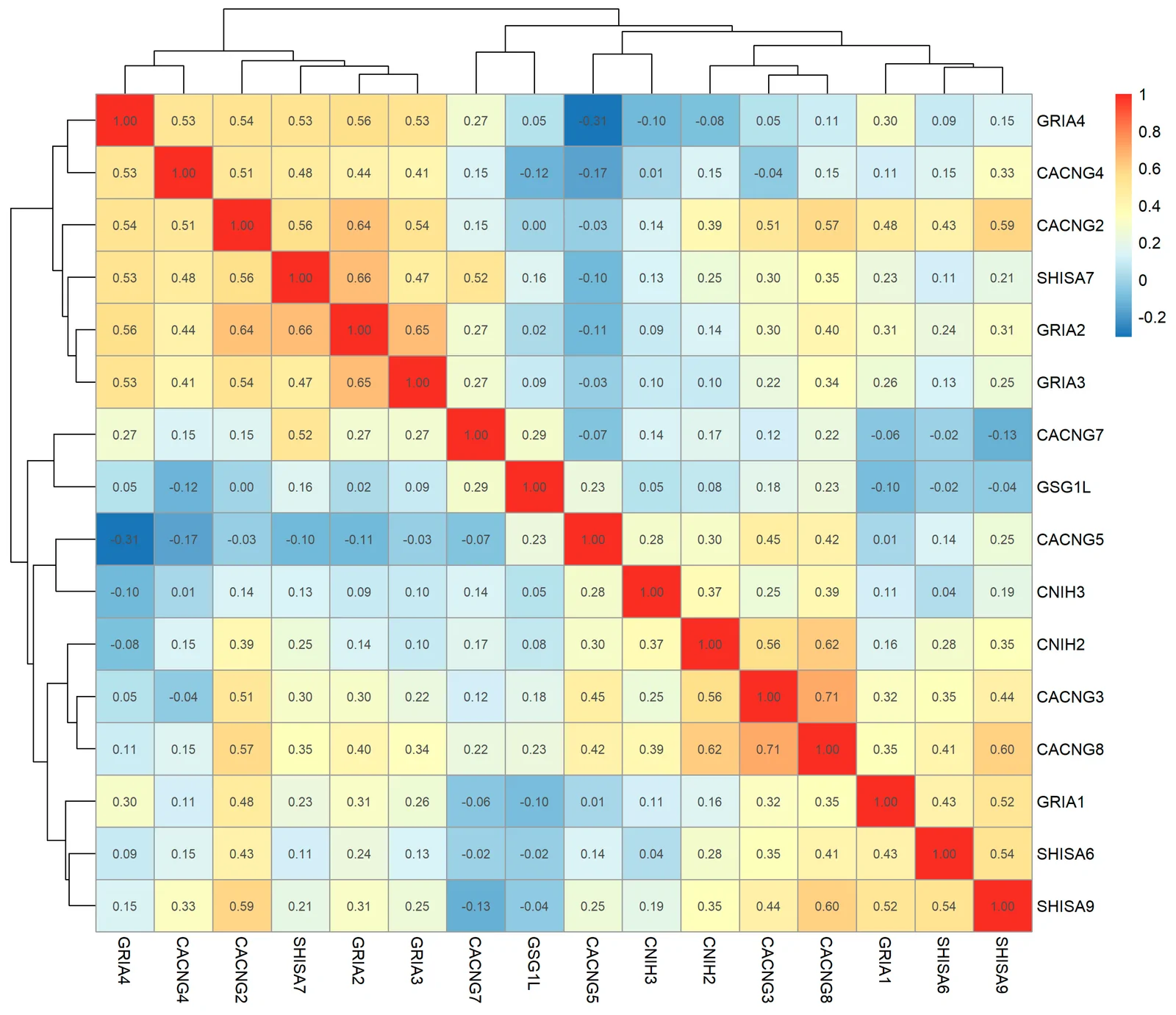
Co-expression of AMPAR subunits and auxiliary proteins in TCGA LGG tumors. Heatmap showing Spearman correlation coefficients between receptor subunit genes *GRIA1*–*GRIA4* and selected auxiliary proteins and interacting partners. Positive correlations are represented by warmer colors and negative correlations by cooler colors.

We next sought to examine the co-expression patterns between *GRIA1*–*GRIA4* and a panel of selected genes encoding synaptic auxiliary proteins including transmembrane AMPAR regulatory proteins (TARPs) [32]. Expression of AMPAR subunit-encoding genes indicated coordinated association with genes coding several established auxiliary proteins. For example, *CACNG2* exhibited overall strong and consistent correlations with all four AMPAR subunits (*r* = 0.48–0.64). Other positive correlations were observed for *CACNG4* and *SHISA9*. These findings suggest that a subset of genes encoding canonical AMPAR auxiliary proteins participates in a coordinated transcriptional program in LGG (Figure 5).

### 3.5. Functional Enrichment Analysis Shows a Synaptic Program Associated with AMPAR Subunit Gene Expression

**Figure 6:**
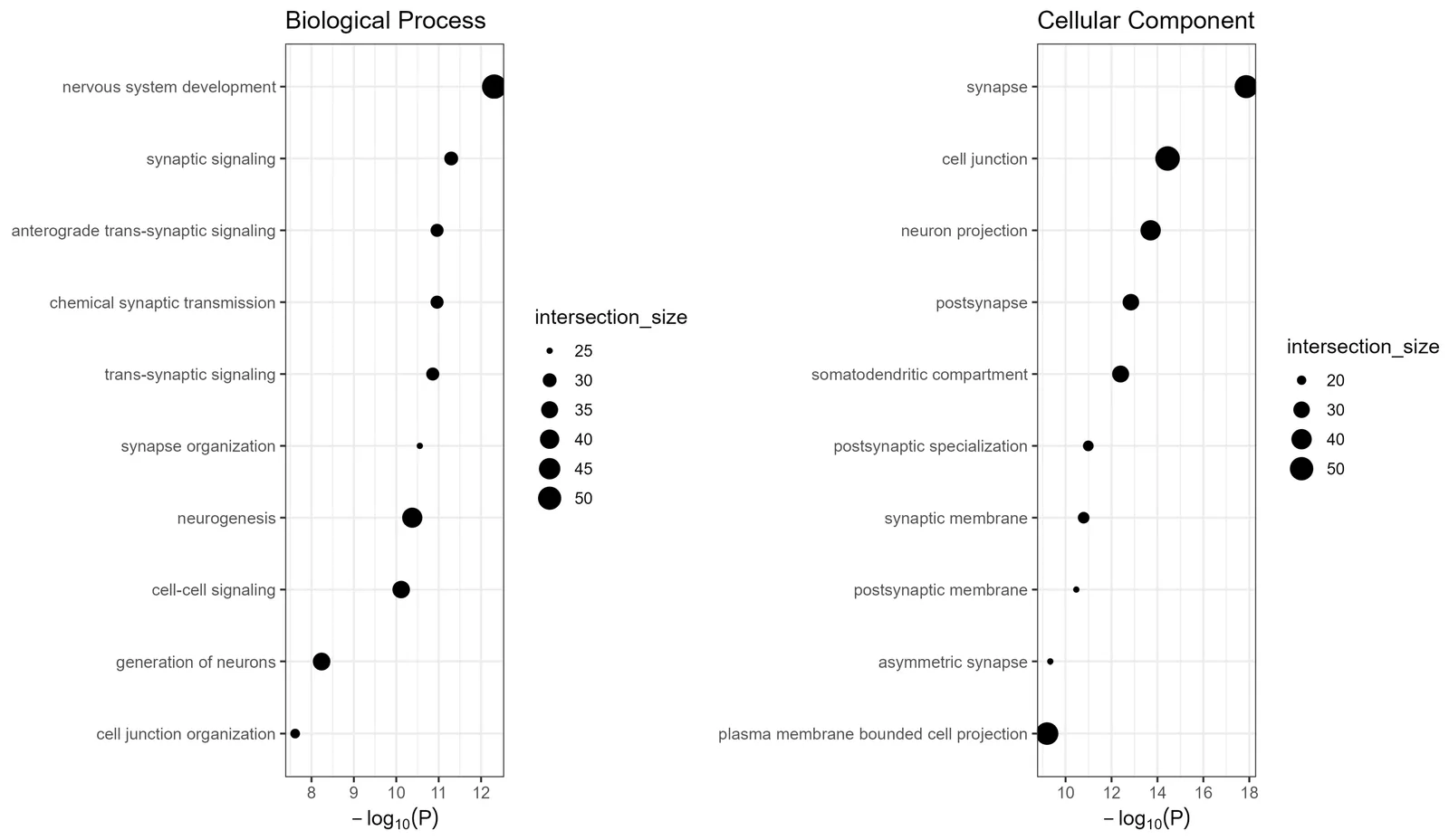
Functional enrichment analysis of genes associated with the AMPAR transcriptional program in TCGA LGG tumors. Gene Ontology enrichment analysis was performed on genes positively correlated with the AMPAR expression score in TCGA-LGG. Left, top enriched Biological Process terms; Right, top enriched Cellular Component terms. Dot size represents the number of genes associated with each GO term (intersection size), and the x-axis indicates enrichment significance expressed as −log10(*p* value).

Gene Ontology analysis demonstrated highly significant enrichment for synapse-related biological processes. The strongest Biological Process terms included synapse organization, chemical synaptic transmission, trans-synaptic signaling, and synaptic signaling. Cellular Component analysis identified synaptic membrane, postsynaptic membrane, synapse, postsynaptic density, and glutamatergic synapse among the most significantly enriched terms. Overall, enriched categories were thus predominantly related to synaptic signaling, trans-synaptic communication, synapse organization, postsynaptic structures, and glutamatergic neurotransmission. These findings support the existence of a coordinated synaptic transcriptional program associated with elevated AMPAR subunit gene expression in LGG (Figure 6; Table 2).

### 3.6. GRIA1–GRIA4 Expression Associates with Better Survival in Patients with LGG

**Figure 7:**
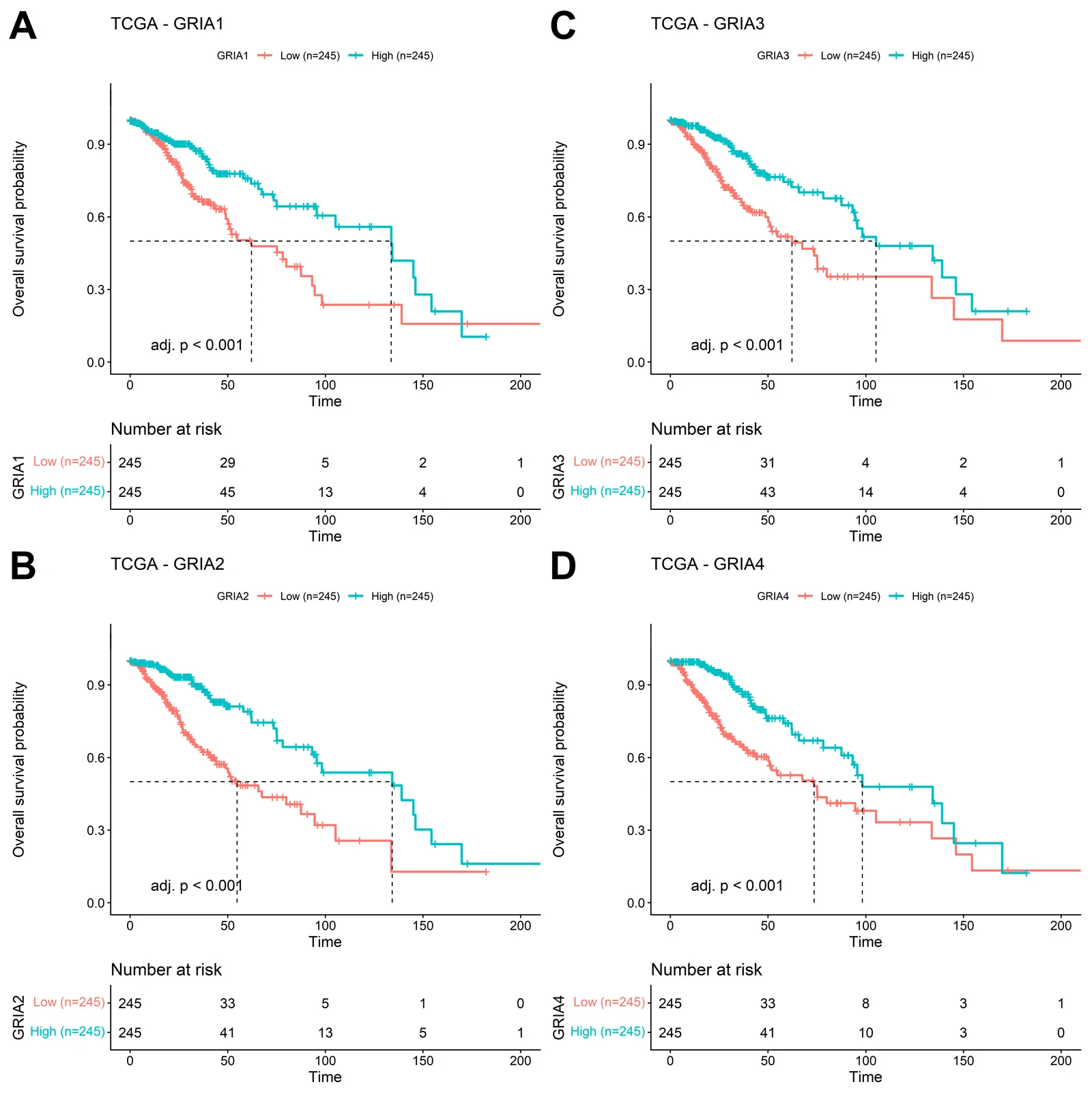
Kaplan–Meier analysis of OS in patients bearing TCGA LGG tumors with higher or lower expression of *GRIA1* (**A**), *GRIA2* (**B**), *GRIA3* (**C**), and *GRIA4* (**D**). The number of samples and BH FDR adjusted *p*-values are indicated in the panels.

**Figure 8:**
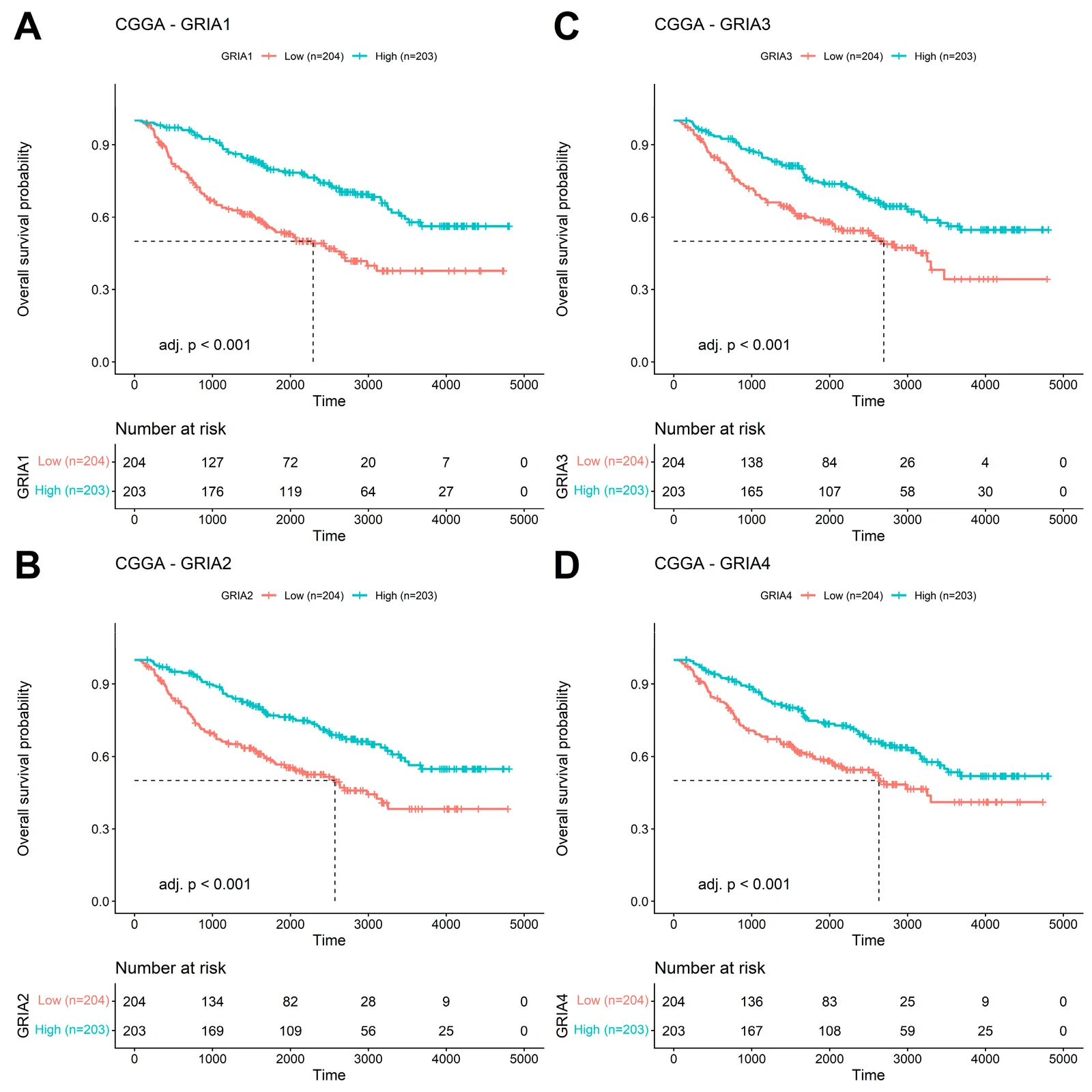
Kaplan–Meier analysis of OS in patients bearing CGGA tumors with higher or lower expression of *GRIA1* (**A**), *GRIA2* (**B**), *GRIA3* (**C**), and *GRIA4* (**D**). The number of samples and BH FDR adjusted *p*-values are indicated in the panels.

High expression levels of *GRIA1*, *GRIA2*, *GRIA3*, or *GRIA4* were significantly associated with longer OS in patients with LGG in both the TCGA (Figure 7) and CGGA (Figure 8) datasets. Because molecular subtype is a major determinant of prognosis in LGG, we additionally performed subtype-stratified Kaplan–Meier analyses within LGG-IDH-mut-codel, LGG-IDH-mut-non-codel, and LGG-IDH-wt. Nominal associations were observed for *GRIA3* (raw *p* = 0.0157) and *GRIA4* (raw *p* = 0.0413) in LGG-IDH-mut-non-codel. However, none of the subtype-specific comparisons remained statistically significant after BH FDR correction (Supplementary Table S1). These findings indicate that the survival associations observed in the overall glioma cohorts should be interpreted in the context of molecular subtype and are consistent with *GRIA* expression marking the favorable neuronal/synaptic transcriptional phenotype enriched in IDH-mutant gliomas rather than serving as an independent prognostic biomarker within individual molecular subgroups.

### 3.7. AMPAR Subunit Gene Expression Associates with More Favorable LGG Biological Types, but Not Independently as Prognostic Factor for Better Outcome

**Figure 9:**
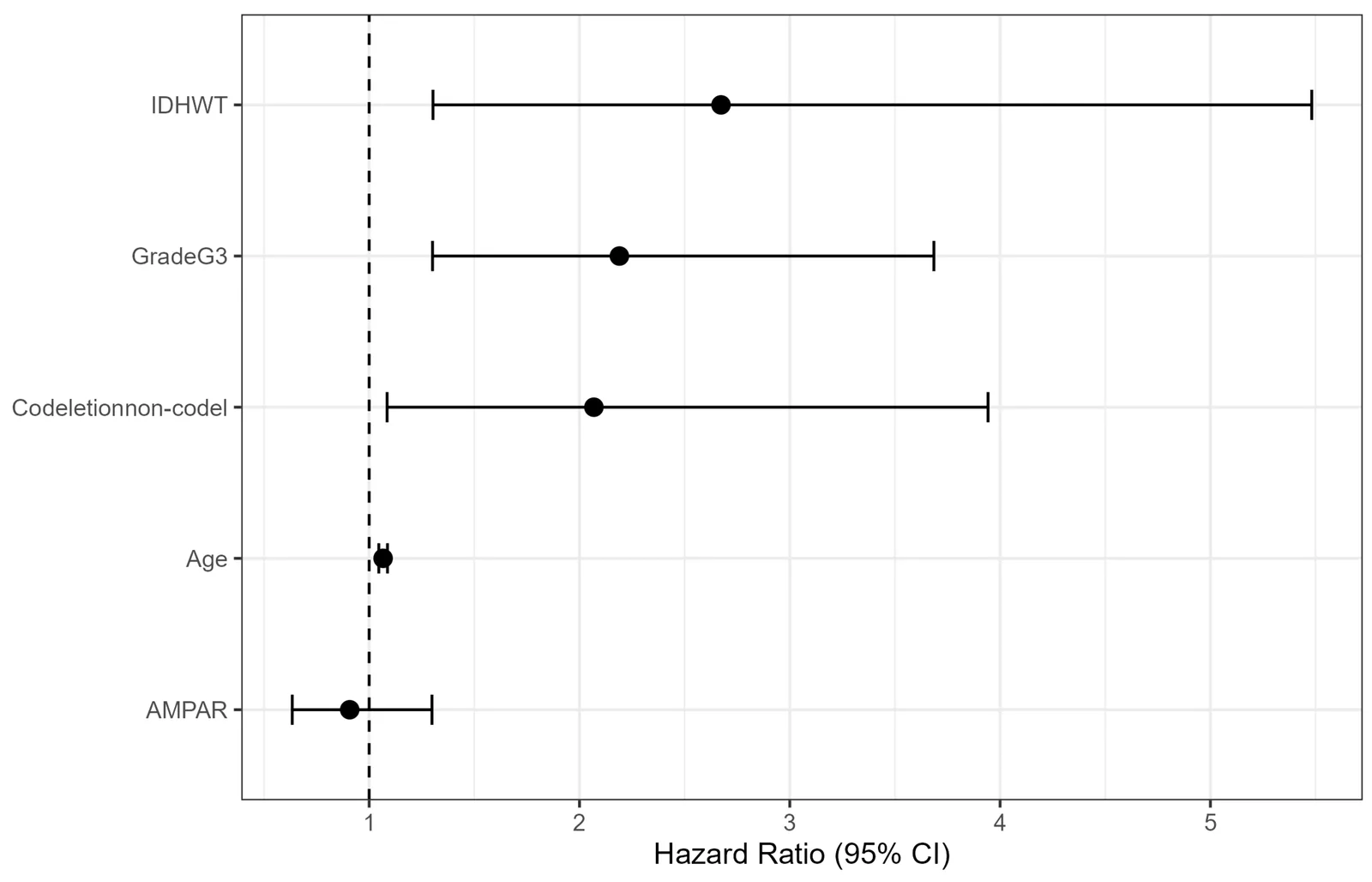
Forest plots showing multivariable Cox proportional hazards analyses for OS in TCGA LGG tumors. Models included AMPAR gene score expression status together with established clinicomolecular prognostic variables, namely IDH mutation status, tumor grade, 1p/19q codeletion status, and age. Hazard ratios (HRs) and 95% confidence intervals are shown. HR < 1 indicates favorable prognostic association, whereas HR > 1 indicates increased risk of death.

**Figure 10:**
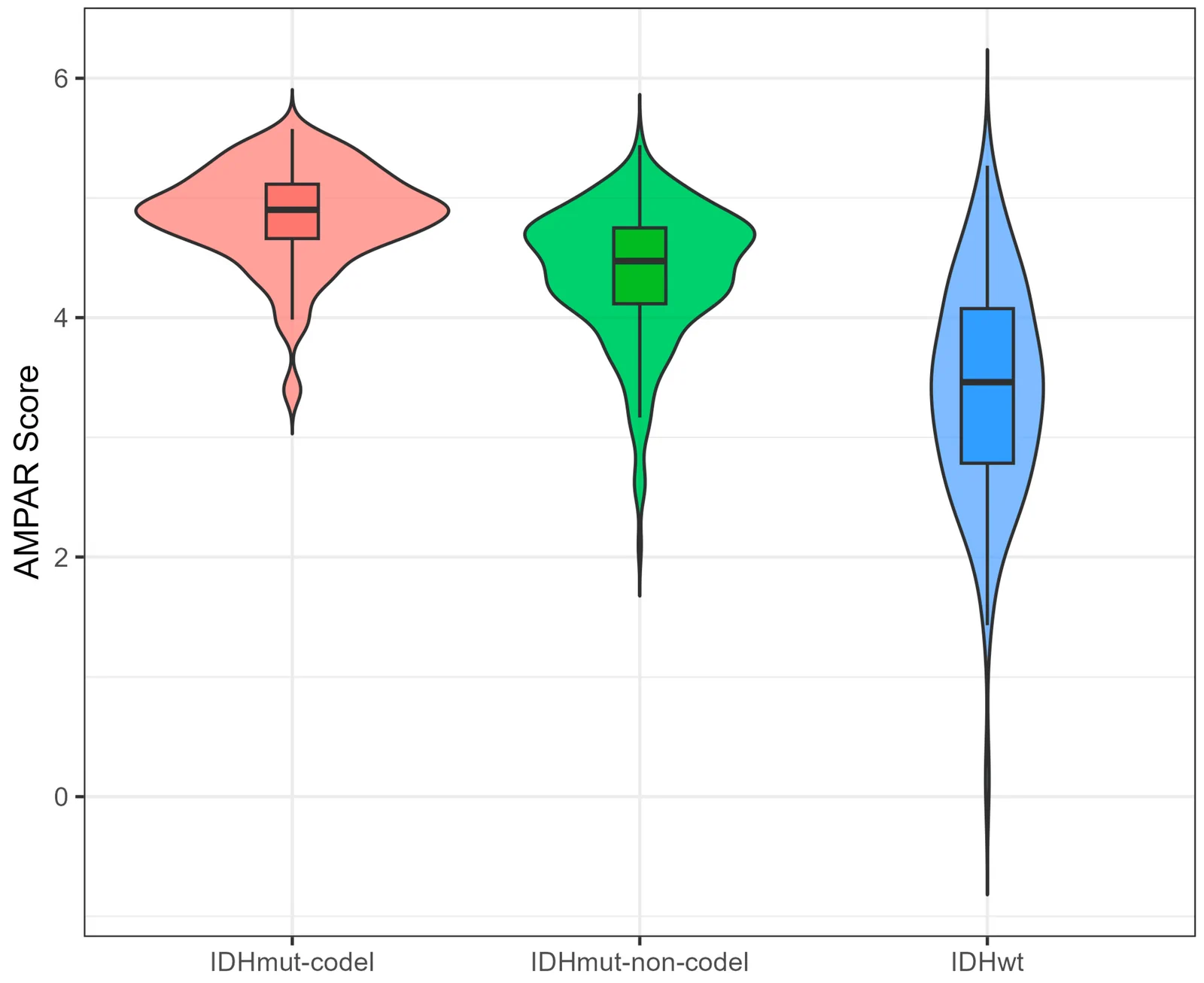
Visualization of the expression levels of the AMPAR gene score in LGG-IDH-mut-codel (*n* = 164), LGG-IDH-mut-non-codel (*n* = 235), and LGG-IDH-wt (*n* = 91) TCGA tumors.

To determine whether the association between *GRIA* expression and OS was independent of established prognostic variables, multivariable Cox regression analyses were performed including age, WHO grade, IDH mutation status, and 1p/19q-codeletion status. The results demonstrated that expression levels of AMPAR subunit-coding genes in TCGA LGG tumors did not retain significant independent prognostic association with OS after adjustment for these clinicomolecular variables. Forest plot visualization showed the relative contribution of each covariate to OS risk. As expected, IDH mutation and 1p/19q codeletion were associated with better survival, whereas increasing age and higher tumor grade were associated with worse outcome. Confidence intervals for expression of the AMPAR gene score crossed the null hazard ratio, indicating lack of statistical significance (Figure 9). Therefore, AMPAR subunit gene expression in TCGA tumors was associated with favorable survival in univariate analyses, but this association was attenuated after adjustment for established clinicomolecular variables, indicating that *GRIA1*–*GRIA4* expression is associated with a favorable LGG biology profile, but is not an independent prognostic factor. In fact, more pronounced expression of any of the four individual *GRIA* genes was observed in LGG-IDH-mut-codel tumors, which have a more favorable prognosis, and the lowest expression occurred in LGG-IDH-wt, which show a worse prognosis, as seen above (Figure 1 and Figure 2). This finding was further supported by comparison among LGG types using the composite AMPAR gene score containing all four genes together (Figure 10).

### 3.8. The GRIA Gene Signature in LGG Is Not Due to Stromal and Immune Infiltration

Given that the gene expression and survival results may be influenced by non-tumoral cells contained within the bulk glioma samples, we next performed a series of analyses to clarify the contribution of different cell types in TCGA tumors. Higher AMPAR scores were significantly associated with lower stromal (*r* = −0.457), immune (*r* = −0.437), and ESTIMATE scores (*r* = −0.463; all *p*s < 2.2 × 10^−16^) (Supplementary Table S2), indicating that LGG tumors with stronger AMPAR signatures exhibited reduced stromal and immune infiltration. These findings indicate that differences in stromal and immune infiltration are unlikely to fully explain the observed AMPAR-associated transcriptional program.

### 3.9. GRIA Gene Expression in LGG Correlates with Neuronal Enrichment

We next carried out a xCell deconvolution analysis in LGG tumors, which demonstrated that the neuronal enrichment score showed the strongest positive association with the AMPAR score (*r* = 0.601), whereas macrophage, dendritic-cell, endothelial, stromal, and immune signatures were inversely associated. Analysis of individual *GRIA* genes showed that all four genes correlated positively with neuronal enrichment, with the strongest association observed for *GRIA2*, followed by *GRIA3*, *GRIA1*, and *GRIA4* (Table 3, Supplementary Table S3).

### 3.10. GRIA Genes Are Highly Expressed in Glioma Malignant Cells Compared to Other Cell Types

**Figure 11:**
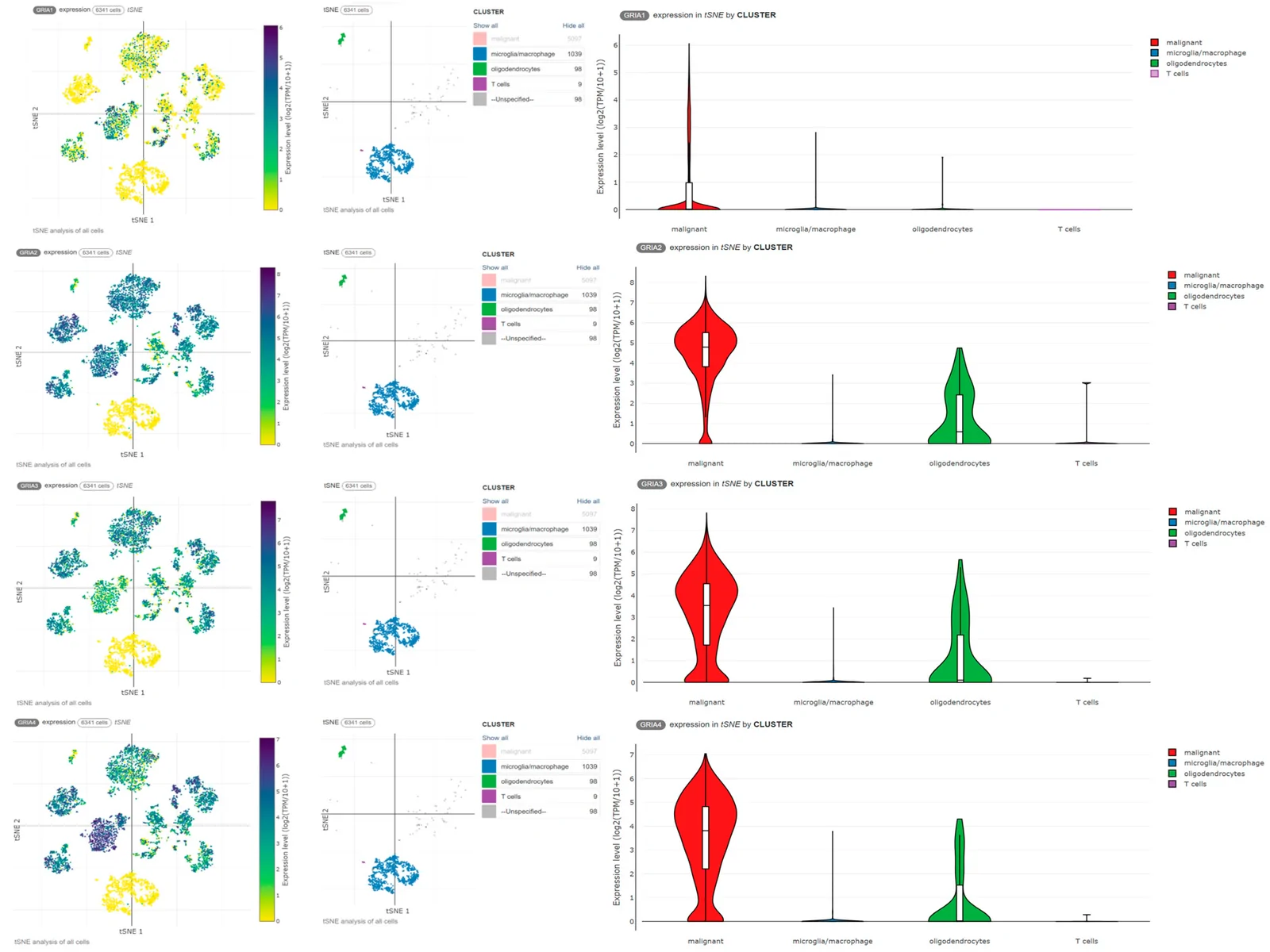
Single-cell RNA-seq analysis of *GRIA1*, *GRIA2*, *GRIA3*, and *GRIA4* expression in SCP50 IDH-mutant astrocytomas. Results are shown for cell types detectable in the SCP50 dataset, namely malignant glioma cells, microglia/macrophages, oligodendrocytes, and T cells.

Analysis of a published single-cell RNA-seq dataset of IDH-mutant astrocytoma demonstrated that *GRIA1*, *GRIA2*, *GRIA3*, and *GRIA4* transcripts were detectable in malignant glioma cells. All four genes showed robust expression within malignant cells, whereas expression in microglia/macrophages and T cells was minimal (Figure 11). Oligodendrocytes also expressed these genes, but generally at lower levels than malignant cells. No neurons are detectable in the SCP50 dataset.

## 4. Discussion

Neuronal activity is increasingly recognized as an important regulator of glioma biology. Studies in GBM have demonstrated that malignant glioma cells can exploit neurotransmitter signaling pathways involved in normal CNS development and plasticity to promote tumor growth and survival [1,9,10,11,33,34]. Among these pathways, glutamatergic signaling mediated by AMPAR has attracted considerable attention because of its role in neuron–glioma communication and tumor progression [4,7,24]. Although expression of *GRIA* genes has previously been proposed as a potential biomarker in glioma [35,36], the biological and clinical significance of AMPAR subunit expression in LGG has remained poorly understood.

In the present study, we show that elevated expression of *GRIA1*, *GRIA2*, *GRIA3*,or *GRIA4* is consistently associated with favorable clinicomolecular characteristics in LGG. Expression of all four AMPAR subunit genes was highest in IDH-mutant/1p19q-codeleted tumors and lowest in IDH-wildtype gliomas, mirroring the established prognostic hierarchy of diffuse glioma molecular subtypes. Furthermore, increased expression of each *GRIA* gene was associated with prolonged patient survival. Together, these findings indicate that AMPAR subunit gene expression is closely linked to a biologically favorable glioma phenotype.

These observations may appear counterintuitive, given that experimental studies have shown that AMPAR-mediated signaling can contribute to glioma growth and invasion, and pharmacological inhibition of AMPAR activity has demonstrated antitumor effects in GBM models. Under this paradigm, one might expect increased expression of AMPAR subunits to be associated with more aggressive disease. However, accumulating evidence suggests that the relationship between AMPAR signaling and glioma biology is complex and context-dependent. Sustained AMPAR-mediated calcium influx can promote excitotoxicity, mitochondrial dysfunction, and apoptosis under certain conditions [19,37]. Consistent with this possibility, the Class I ampakine CX614, which enhances AMPAR activity by increasing agonist binding affinity, has been reported to reduce glioblastoma cell viability and induce apoptosis in multiple cancer cell types [38]. Although the present study does not directly assess receptor function, these observations suggest that increased AMPAR expression should not automatically be interpreted as evidence of a tumor-promoting phenotype.

Importantly, our transcriptome-wide analyses indicate that the biological significance of elevated *GRIA* gene expression extends beyond the receptor subunits themselves. Genes positively correlated with the AMPAR expression score included multiple proteins involved in synaptic organization, neuronal connectivity, and AMPAR-associated excitatory transmission and signaling. Functional enrichment analysis demonstrated striking overrepresentation of synapse-related GO categories, including synaptic signaling, chemical synaptic transmission, trans-synaptic signaling, synapse organization, synaptic membrane, postsynaptic membrane, and postsynaptic density. Rather than identifying isolated changes in expression of individual receptor genes, these findings support the existence of a coordinated synaptic transcriptional program associated with elevated AMPAR subunit expression in LGG.

To further examine whether the AMPAR-associated transcriptional program primarily reflected differences in glioma molecular subtype, we performed two complementary analyses. First, statistical adjustment for molecular subtype largely preserved the neuronal/synaptic co-expression pattern observed in the overall cohort. Second, independent transcriptome-wide co-expression analyses performed within each molecular subtype and tumor grade consistently identified neuronal and synaptic transcriptional programs, although the specific genes most strongly associated with *GRIA1*–*GRIA4* gene expression varied across subtypes. These findings indicate that molecular subtype and tumor grade influence the composition of the associated co-expression network but do not fully account for the overall neuronal/synaptic transcriptional program associated with AMPAR expression in LGG. At the same time, these findings do not establish that the observed signature is completely independent of molecular subtype or that it originates exclusively from malignant glioma cells. Rather, they support the interpretation that elevated *GRIA* expression is associated with a robust neuronal/synaptic transcriptional state that remains detectable across clinically relevant glioma subtypes while acknowledging the influence of tumor molecular context.

**Figure 12:**
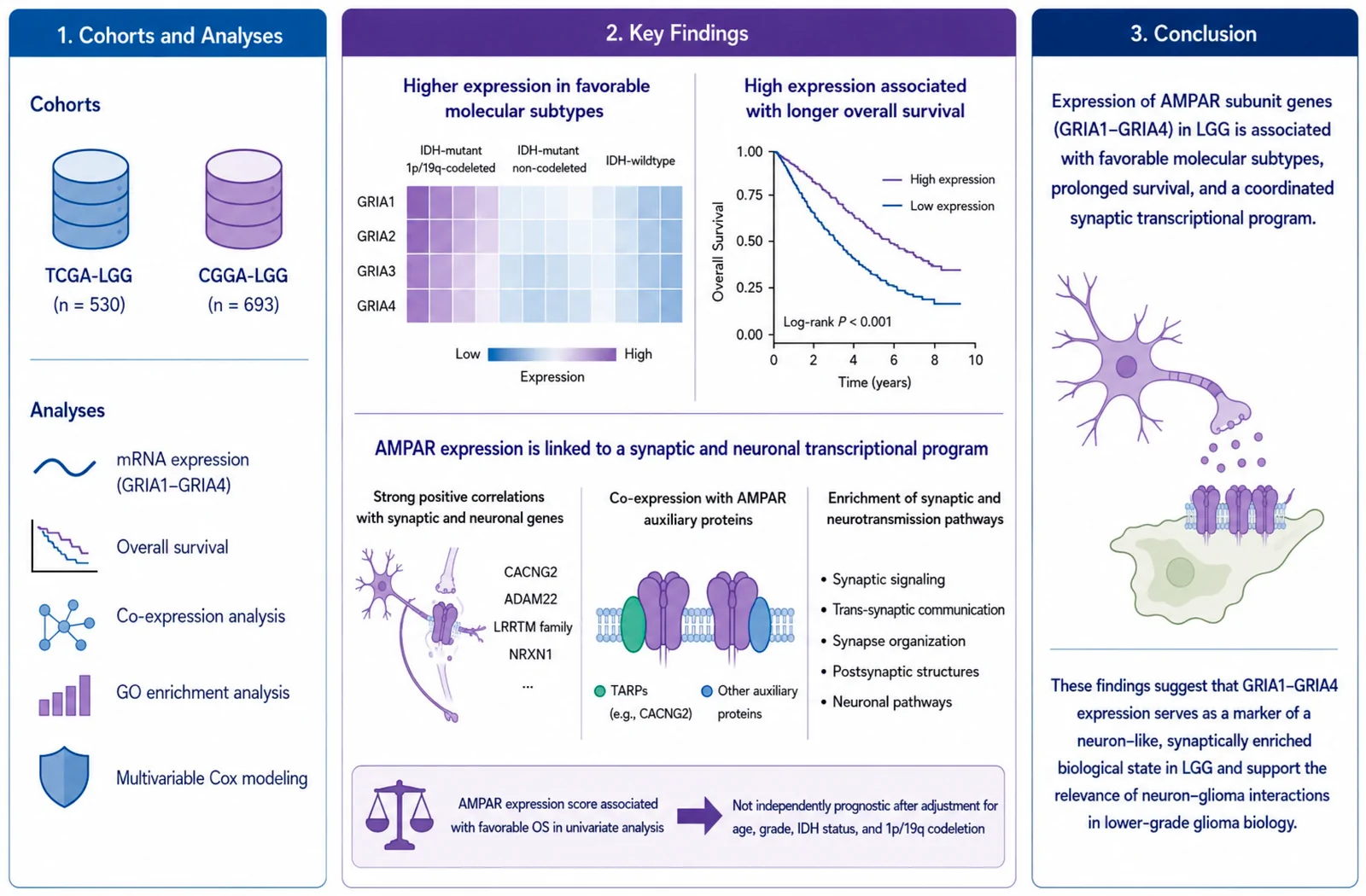
Graphical summary of the study findings. Expression of *GRIA1*–*GRIA4*, which encode AMPAR subunits, is associated with favorable molecular, biological, and clinical features in LGG, suggesting that activation of an excitatory synapse gene program might mark a synaptically enriched transcriptional signature associated with favorable clinicomolecular characteristics and improved prognosis.

The possibility of a consistent synaptic transcriptional program in LGG is further supported by the coordinated expression of AMPAR auxiliary proteins and synaptic organizers identified in our analyses. The strong association between AMPAR subunits and genes involved in synaptic architecture suggests that elevated GRIA expression may serve as a marker of a broader synaptic transcriptional program observed in bulk LGG transcriptomes. We previously reported that increased expression of *DLG2*, *DLG3*, and *DLG4*, which encode the postsynaptic scaffolding proteins PSD-93, SAP-102, and PSD-95, respectively, is associated with improved survival in LGG [39]. The present findings extend this concept by demonstrating that favorable prognosis is associated not only with expression of individual synaptic genes but also with a larger transcriptional network enriched for neuronal and synaptic functions. Figure 12 summarizes a working model in which high expression of excitatory synaptic genes identifies a favorable synaptic transcriptional program in LGG. Whether this signature reflects intrinsic tumor-cell biology, contributions from surrounding neural elements, or both remains to be determined.

One possible interpretation is that tumors exhibiting high expression of synaptic and neuronal genes retain features of a more differentiated cellular phenotype. Consistent with this hypothesis, recent studies have demonstrated that grade 3 astrocytoma and oligodendroglioma cells can be experimentally reprogrammed toward neuron-like states through exposure to small molecules or neural transcription factors, resulting in reduced proliferation, decreased expression of tumor-associated genes, and activation of tumor-suppressive programs [40]. Although the present data do not establish a causal relationship between neuronal differentiation and patient outcome, they support the possibility that preservation of neuronal identity may be linked to less aggressive biological behavior in LGG. This raises the intriguing possibility that therapeutic strategies aimed at promoting differentiation may merit further investigation in diffuse gliomas.

A major concern when interpreting neuronal gene signatures in bulk glioma transcriptomic datasets is that they may simply reflect varying proportions of non-neoplastic neural cells. To address this possibility, we incorporated complementary analyses examining tumor composition. ESTIMATE and xCell demonstrated that tumors with high AMPAR subunit gene expression exhibited reduced stromal and immune infiltration while maintaining a strong neuronal transcriptional signature. Importantly, interrogation of an independent single-cell RNA-seq dataset confirmed that all four *GRIA* genes are expressed by malignant glioma cells themselves. Importantly, although the single-cell analyses demonstrate that malignant glioma cells can express *GRIA* genes, the present study cannot determine the relative contributions of malignant and non-neoplastic neural cells to the bulk RNA-sequencing signal. Therefore, our findings should be interpreted as identifying a tumor-level neuronal/synaptic transcriptional signature rather than assigning its cellular origin. Together, these complementary analyses indicate that stromal and immune infiltration alone are unlikely to account for the observed AMPAR-associated transcriptional signature. Although single-cell analyses demonstrate that malignant glioma cells can express *GRIA* genes, the present data do not permit definitive quantification of the relative contributions of malignant cells and non-neoplastic neural elements to the bulk RNA-sequencing signal.

Several limitations should be acknowledged. First, the TCGA-LGG cohort was generated before the WHO 2021 classification. Consequently, the IDH-wild-type subgroup is molecularly heterogeneous and includes tumors that would now be classified differently under current integrated diagnostic criteria. Second, gene expression does not necessarily reflect protein abundance or receptor activity [41,42,43]. Third, the biological functions of AMPAR signaling in LGG were inferred from transcriptomic associations rather than direct experimental measurements, and therefore cannot establish causality. Additional studies integrating single-cell analyses, proteomic, functional, and mechanistic approaches will be required to determine whether the synaptic transcriptional program identified here actively contributes to LGG behavior or reflects underlying cellular differentiation states. Finally, because these analyses were performed primarily using bulk transcriptomic datasets, the cellular origin of the observed synaptic transcriptional signature cannot be determined definitively.

## 5. Conclusions

The present study demonstrates that expression of the AMPAR subunit genes *GRIA1*, *GRIA2*, *GRIA3*, and *GRIA4* is strongly associated with the molecular landscape of LGG. Elevated expression of these genes was consistently observed in IDH-mutant/1p19q-codeleted tumors, whereas the lowest expression levels occurred in IDH-wildtype tumors. Increased *GRIA* expression was associated with longer OS and with a transcriptional network enriched for synaptic signaling, neuronal connectivity, and AMPAR signaling-associated genes. Functional enrichment analyses further revealed that genes associated with the AMPAR program are concentrated in pathways related to synaptic communication and postsynaptic organization. Although the prognostic association of the AMPAR score was not independent of established clinicomolecular factors, the findings indicate that AMPAR subunit gene expression is consistently associated with favorable glioma biology and a synaptically enriched transcriptional signature. These results may help to extend current understanding of neuron–glioma interactions in LGG and identify AMPAR-associated transcriptional signatures as candidate biomarkers to be integrated in the characterization of biologically distinct glioma subgroups. Our findings identify a tumor-level neuronal and synaptic transcriptional signature associated with AMPAR subunit gene expression, but they do not establish the relative contributions of malignant and non-neoplastic neural cells to the bulk transcriptomic signal.

## Figures and Tables

**Table 1:** Structures and chromosome locations for *GRIA* genes encoding AMPAR subunits.

Gene	Chromosome	Cytogenetic Band	NCBI Gene ID
*GRIA1*	5	5q33.2	2890
*GRIA2*	4	4q32.1	2891
*GRIA3*	X	Xq25	2892
*GRIA4*	11	11q22.3	2893

NCBI, National Center for Biotechnology Information.

**Table 2:** Gene Ontology enrichment analysis of genes positively correlated with *GRIA1*–*GRIA4* expression score in TCGA LGG tumors.

**Biological Process**	
**Term**	***p*** **-Value**
Synaptic signaling	5.1 × 10^−12^
Anterograde trans-synaptic signaling	1.1 × 10^−11^
Chemical synaptic transmission	1.1 × 10^−11^
Trans-synaptic signaling	1.4 × 10^−11^
Synapse organization	2.8 × 10^−11^
**Cellular Component**	
**Term**	***p*** **-Value**
Synapse	1.4 × 10^−18^
Postsynapse	1.4 × 10^−13^
Synaptic membrane	1.6 × 10^−11^
Postsynaptic membrane	3.4 × 10^−11^
Postsynaptic density	2.3 × 10^−9^

Gene Ontology enrichment was performed using g:Profiler on genes positively correlated with the AMPAR expression score. The table reports GO category, GO term name, and adjusted *p* value for each enriched term. Results are shown for Biological Process (GO:BP) and Cellular Component (GO:CC) ontologies.

**Table 3:** Correlations between *GRIA1*–*GRIA4* expression and a neuronal enrichment score in a cell-type deconvolution analysis of TCGA LGG tumors.

Gene	Neuron *r*	Adjusted *p*-Value
GRIA1	0.443	1.27 × 10^−25^
GRIA2	0.591	3.22 × 10^−49^
GRIA3	0.479	3.18 × 10^−30^
GRIA4	0.383	5.60 × 10^−19^

## Data Availability

The original data presented in the study are openly available in The Cancer Genome Atlas (TCGA) Research Network (https://www.cancer.gov/tcga), the Chinese Glioma Genome Atlas (CGGA, http://www.cgga.org.cn), and the Broad Institute Single Cell Portal (https://singlecell.broadinstitute.org/single_cell/study/SCP50/single-cell-rna-seq-analysis-of-astrocytoma).

## Supplementary Materials

The following supporting information can be downloaded at: https://www.mdpi.com/article/10.3390/brainsci16080773/s1, Supplementary Figure S1: Within-subtype transcriptome-wide co-expression analyses of the AMPAR-associated transcriptional program in LGG; Supplementary Figure S2: Transcriptome-wide co-expression analysis of AMPAR subunit genes after statistical adjustment for molecular subtype in LGG; Supplementary Figure S3 Within-grade transcriptome-wide co-expression analyses of the AMPAR-associated transcriptional program in LGG; Supplementary Table S1: FDR-adjusted p-values for subtype-stratified Kaplan–Meier survival analyses of GRIA1–GRIA4 expression in TCGA LGG; Supplementary Table S2: Correlations between AMPAR expression score and ESTIMATE-derived stromal, immune, and ESTIMATE scores in TCGA LGG; Supplementary Table S3: Top positively and negatively correlated xCell cell-type enrichment scores for *GRIA1*–*GRIA4* expression in TCGA LGG.

## Abbreviations

The following abbreviations are used in this manuscript:

AMPA	α-amino-3-hydroxy-5-methyl-4-isoxazolepropionic acid
AMPAR	α-amino-3-hydroxy-5-methyl-4-isoxazolepropionic acid receptor
BH	Benjamini–Hochberg
BP	Biological Process
CC	Cellular Component
CGGA	Chinese Glioma Genome Atlas
CNS	False Discovery Rate
FDR	Central nervous system
GBM	Glioblastoma
GO	Gene Ontology
HR	Hazard Ratio
IDH	Isocitrate dehydrogenase
LGG	Lower-grade glioma
LTP	Long-term potentiation
mut-codel	Mutant with 1p/19q codeletion
mut-non-codel	Mutant without 1p/19q codeletion
NCBI	National Center for Biotechnology Information
OS	Overall survival
TCGA	The Cancer Genome Atlas
WHO	World Health Organization
wt	Wildtype
