# Supplementary material for: AMPAR Subunit Gene Expression Marks a Synaptic Transcriptional State in Lower-Grade Glioma
Source: Brain Sci. 2026 Jul 23;16(8):773. doi: 10.3390/brainsci16080773 (PMC13510890; doi:10.3390/brainsci16080773)
Supplement: Supplementary file 1 [file brainsci-16-00773-s001.zip › brainsci-4414488-supplementary.pdf]

Supplementary Information for

AMPAR Subunit Gene Expression Marks a Synaptic Transcriptional State in Lower-Grade Glioma

Bruno Rodrigues • Matheus Dalmolin • Henrique Ritter Dal-Pizzol • Osvaldo Malafaia • Marcelo A.C. Fernandes • Karina Munhoz de Paula Alves Coelho • Rafael Roesler • Gustavo R. Isolan

Supplementary Table S1. FDR-adjusted p-values for subtype-stratified Kaplan–Meier survival analyses of GRIA1–GRIA4 expression in TCGA LGG.

Gene	Glioma subtype	*n*	FDR-adjusted *p*-vale
GRIA1	IDH-mut-codel	164	0.8500
	IDH-mut-non-codel	235	0.2652
	IDH-wt	91	0.8500
GRIA2	IDH-mut-codel	164	0.8500
	IDH-mut-non-codel	235	0.4839
	IDH-wt	91	0.8500
GRIA3	IDH-mut-codel	164	0.9509
	IDH-mut-non-codel	235	0.1884
	IDH-wt	91	0.8500
GRIA4	IDH-mut-codel	164	0.8500
	IDH-mut-non-codel	235	0.2478
	IDH-wt	91	0.8500

FDR, False Discovery Rate.

Supplementary Table S2. Correlations between AMPAR expression score and ESTIMATE-derived stromal, immune, and ESTIMATE scores in TCGA LGG.

**Variable**	**Spearman *r***	***p*-value**
Stromal score	−0.457	$< 2.2\times10^{-16}$
Immune score	−0.437	$< 2.2\times10^{-16}$
ESTIMATE score	−0.463	$<2.2\times10^{-16}$

Supplementary Table S3. Top positively and negatively correlated xCell cell-type enrichment scores for *GRIA1–GRIA4* expression in TCGA LGG.

	Gene	CellType	Rho	P	FDR
55	GRIA1	Platelets	0.1271960	3.236014e-03	7.108622e-03
45	GRIA1	Myocytes	0.1403419	1.147620e-03	2.746091e-03
34	GRIA1	Macrophages M2	0.1420782	9.939705e-04	2.421674e-03
22	GRIA1	Eosinophils	0.1464528	6.870712e-04	1.689313e-03
12	GRIA1	CD8+ T-cells	0.1472297	6.427727e-04	1.625124e-03
6	GRIA1	CD4+ memory T-cells	0.1895017	1.039271e-05	2.994887e-05
64	GRIA1	Tregs	0.2305152	7.161098e-08	2.864439e-07
52	GRIA1	pDC	0.2422805	1.425479e-08	6.262758e-08
56	GRIA1	Preadipocytes	0.4142269	1.485288e-23	2.487857e-22
47	GRIA1	Neurons	0.4431791	4.250611e-27	1.265737e-25

	Gene	CellType	Rho	P	FDR
120	GRIA2	Pericytes	0.1470935	6.503436e-04	1.628898e-03
89	GRIA2	Eosinophils	0.1629343	1.558021e-04	4.053879e-04
73	GRIA2	CD4+ memory T-cells	0.2011613	2.791235e-06	8.598287e-06
79	GRIA2	CD8+ T-cells	0.2510331	4.057469e-09	1.912900e-08
112	GRIA2	Myocytes	0.3218137	2.480396e-14	2.374093e-13
121	GRIA2	Plasma cells	0.3419465	4.304503e-16	4.806695e-15
131	GRIA2	Tregs	0.3775144	1.566941e-19	1.908819e-18
119	GRIA2	pDC	0.3968916	1.362426e-21	1.921738e-20
123	GRIA2	Preadipocytes	0.4247672	8.342639e-25	1.597019e-23
114	GRIA2	Neurons	0.5912327	1.200457e-51	3.217224e-49

	Gene	CellType	Rho	P	FDR
156	GRIA3	Eosinophils	0.1021575	1.820856e-02	3.500939e-02
187	GRIA3	Pericytes	0.1689690	8.719666e-05	2.360475e-04
146	GRIA3	CD8+ T-cells	0.2151836	5.171790e-07	1.776974e-06
140	GRIA3	CD4+ memory T-cells	0.2636218	6.116706e-10	3.152456e-09
198	GRIA3	Tregs	0.2676126	3.286941e-10	1.761800e-09
188	GRIA3	Plasma cells	0.2908241	7.210228e-12	4.954721e-11
186	GRIA3	pDC	0.3012138	1.159953e-12	8.635208e-12
179	GRIA3	Myocytes	0.3163283	7.111477e-14	5.775382e-13
190	GRIA3	Preadipocytes	0.4285361	2.906282e-25	6.490697e-24
181	GRIA3	Neurons	0.4788081	5.940809e-32	3.184273e-30

	Gene	CellType	Rho	P	FDR
240	GRIA4	MEP	0.2303491	7.321720e-08	2.885619e-07
213	GRIA4	CD8+ T-cells	0.2334551	4.822456e-08	2.019403e-07
254	GRIA4	Pericytes	0.2856521	1.742062e-11	1.037495e-10
253	GRIA4	pDC	0.2881208	1.145963e-11	7.312333e-11
207	GRIA4	CD4+ memory T-cells	0.2898259	8.560524e-12	5.735551e-11
212	GRIA4	CD8+ naive T-cells	0.3204965	3.200542e-14	2.884800e-13
255	GRIA4	Plasma cells	0.3387435	8.370652e-16	8.973339e-15
248	GRIA4	Neurons	0.3828400	4.388223e-20	5.600208e-19
257	GRIA4	Preadipocytes	0.4347864	4.909545e-26	1.196144e-24
246	GRIA4	Myocytes	0.4928819	4.935518e-34	3.306797e-32

	Gene	CellType	Rho	P	FDR
62	GRIA1	Th1 cells	-0.3065495	4.409182e-13	3.475473e-12
35	GRIA1	Mast cells	-0.2962765	2.789520e-12	2.020518e-11
44	GRIA1	mv Endothelial cells	-0.2510146	4.068482e-09	1.912900e-08
67	GRIA1	MicroenvironmentScore	-0.2463905	7.948900e-09	3.599436e-08
15	GRIA1	cDC	-0.2267913	1.172732e-07	4.305370e-07

66	GRIA1	StromaScore	-0.2216034	2.299136e-07	8.215578e-07
21	GRIA1	Endothelial cells	-0.2119299	7.726996e-07	2.588544e-06
40	GRIA1	Mesangial cells	-0.2053746	1.702446e-06	5.367711e-06
33	GRIA1	Macrophages M1	-0.2017193	2.615877e-06	8.151803e-06
5	GRIA1	Basophils	-0.2005279	3.003945e-06	9.148378e-06

	Gene	CellType	Rho	P	FDR
85	GRIA2	CLP	-0.5369371	3.293305e-41	4.413029e-39
90	GRIA2	Epithelial cells	-0.5326856	1.806478e-40	1.613787e-38
99	GRIA2	Macrophages	-0.4587460	3.780096e-29	1.266332e-27
100	GRIA2	Macrophages M1	-0.4158092	9.702623e-24	1.733535e-22
82	GRIA2	cDC	-0.4089917	5.979262e-23	8.902457e-22
134	GRIA2	MicroenvironmentScore	-0.3957049	1.838610e-21	2.463738e-20
133	GRIA2	StromaScore	-0.3288348	6.240884e-15	6.432911e-14
132	GRIA2	ImmuneScore	-0.3204502	3.229254e-14	2.884800e-13
95	GRIA2	HSC	-0.3176492	5.529229e-14	4.630730e-13
88	GRIA2	Endothelial cells	-0.2933145	4.684520e-12	3.303819e-11

	Gene	CellType	Rho	P	FDR
157	GRIA3	Epithelial cells	-0.4633779	8.842629e-30	3.949708e-28
152	GRIA3	CLP	-0.4354459	4.060820e-26	1.088300e-24
166	GRIA3	Macrophages	-0.3231978	1.895026e-14	1.880988e-13
167	GRIA3	Macrophages M1	-0.3044968	6.411826e-13	4.909627e-12
162	GRIA3	HSC	-0.2864461	1.523199e-11	9.277669e-11
149	GRIA3	cDC	-0.2847673	2.022205e-11	1.178154e-10
201	GRIA3	MicroenvironmentScore	-0.2771471	7.147547e-11	4.075622e-10
199	GRIA3	ImmuneScore	-0.2678320	3.175682e-10	1.761800e-09
169	GRIA3	Mast cells	-0.2677244	3.229783e-10	1.761800e-09
177	GRIA3	MSC	-0.2654062	4.639500e-10	2.438012e-09

	Gene	CellType	Rho	P	FDR
224	GRIA4	Epithelial cells	-0.4589182	3.582791e-29	1.266332e-27
219	GRIA4	CLP	-0.4267205	4.838095e-25	9.973920e-24
244	GRIA4	MSC	-0.4093498	5.440073e-23	8.576116e-22
238	GRIA4	Melanocytes	-0.3586695	1.177072e-17	1.371545e-16
235	GRIA4	Macrophages M2	-0.3182107	4.966344e-14	4.293485e-13
233	GRIA4	Macrophages	-0.2874453	1.285641e-11	8.012834e-11
268	GRIA4	MicroenvironmentScore	-0.2495975	5.002572e-09	2.311533e-08
266	GRIA4	ImmuneScore	-0.2394607	2.115200e-08	9.143124e-08
229	GRIA4	HSC	-0.2376797	2.707051e-08	1.151571e-07
211	GRIA4	CD4+ Tem	-0.2294532	8.249830e-08	3.114021e-07

	Gene	CellType	Rho	P	FDR
47	GRIA1	Neurons	0.4431791	4.250611e-27	1.265737e-25
114	GRIA2	Neurons	0.5912327	1.200457e-51	3.217224e-49
181	GRIA3	Neurons	0.4788081	5.940809e-32	3.184273e-30
248	GRIA4	Neurons	0.3828400	4.388223e-20	5.600208e-19

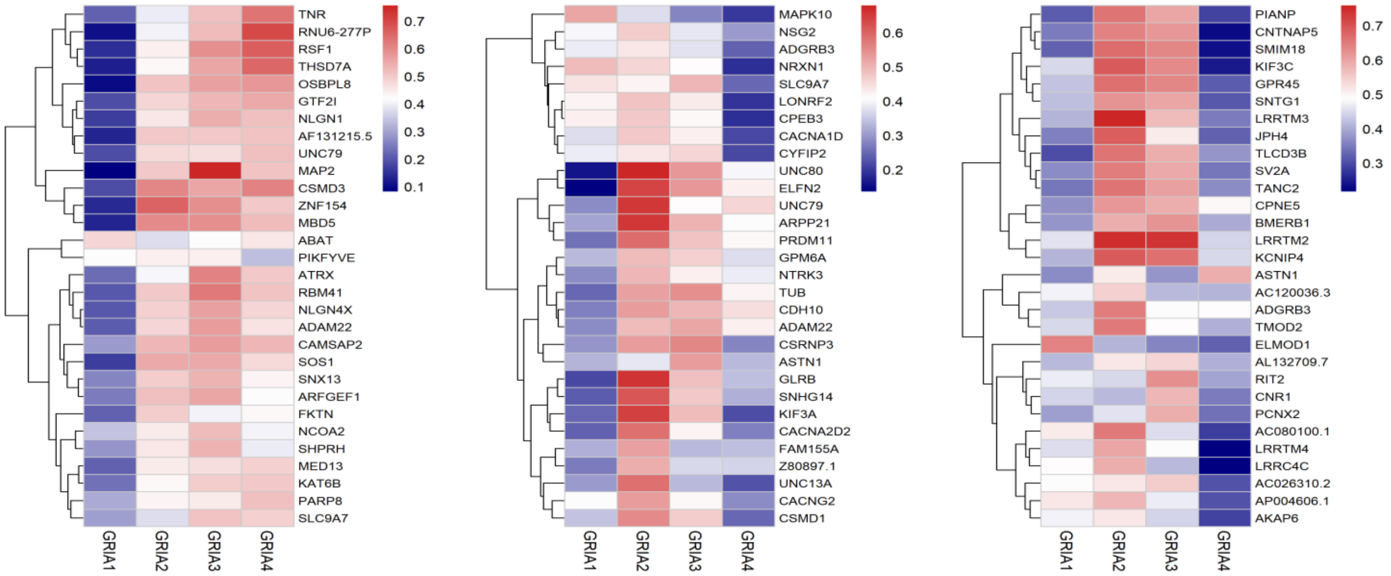

Supplementary Figure S1. Within-subtype transcriptome-wide co-expression analyses of the AMPAR-associated transcriptional program in LGG. Transcriptome-wide Spearman correlation analyses were performed independently within IDH-mut- codel (left panel), IDH-mut-non-codel (center panel), and IDH-wt (right panel) tumors. For each molecular subtype, an AMPAR expression score was calculated as the mean expression of *GRIA1*, *GRIA2*, *GRIA3*, and *GRIA4*, and genes were ranked according to their Spearman correlation with the subtype-specific AMPAR score. The 30 genes showing the strongest positive correlations are displayed together with their correlations with the individual AMPAR subunit genes.

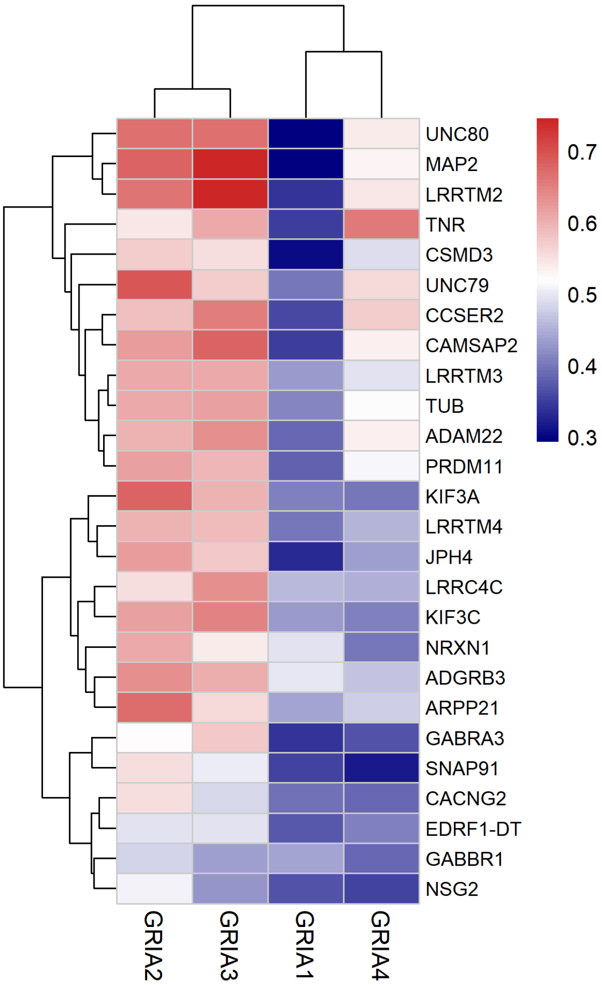

Supplementary Figure S2. Transcriptome-wide co-expression analysis of AMPAR subunit genes after statistical adjustment for molecular subtype in LGG. Transcriptome-wide Spearman correlation analysis between the AMPAR expression score (mean expression of *GRIA1*, *GRIA2*, *GRIA3*, and *GRIA4*) and all expressed genes following statistical adjustment of gene expression values for molecular subtype. The 30 genes showing the strongest positive correlations with the subtype-adjusted AMPAR score are displayed together with their correlations with the individual AMPAR subunit genes.

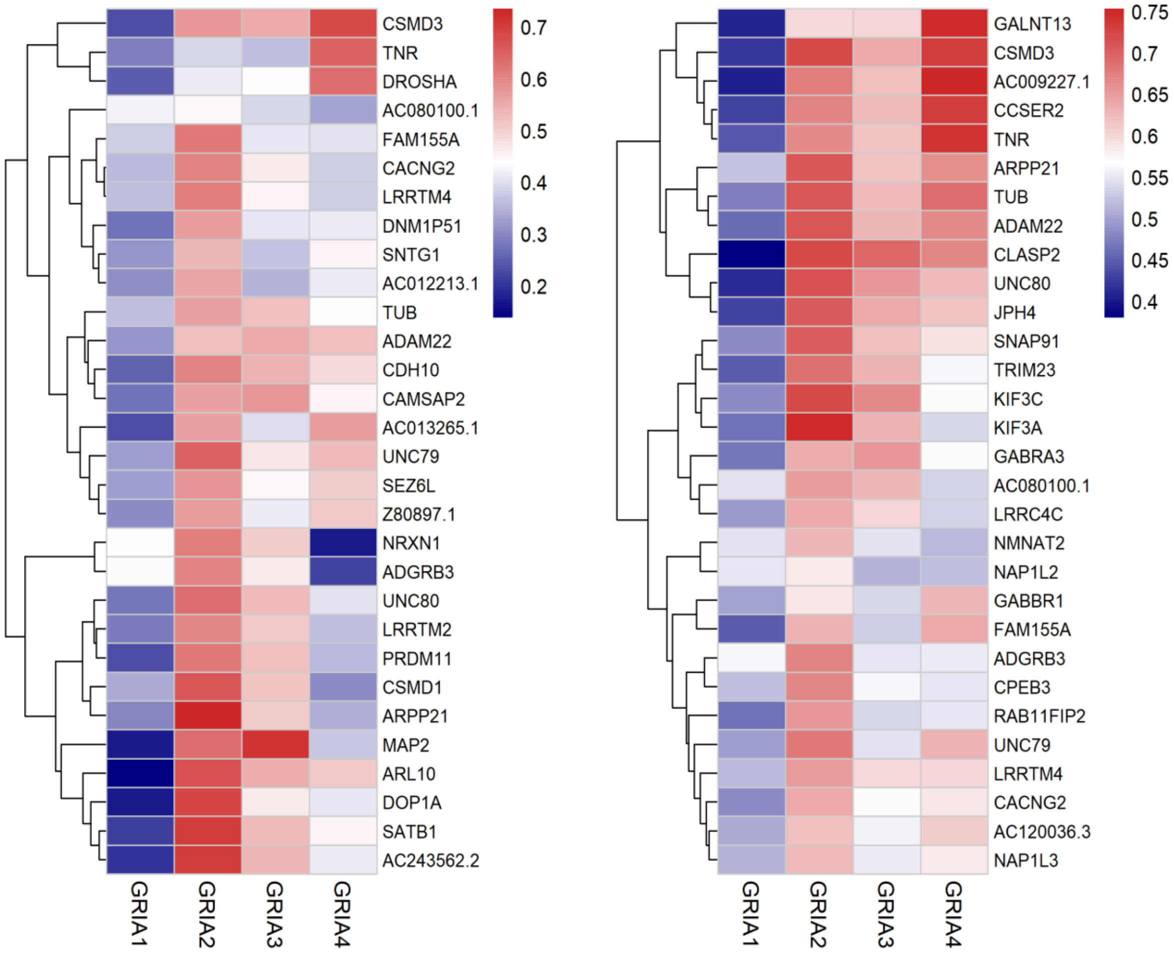

Supplementary Figure S3. Within-grade transcriptome-wide co-expression analyses of the AMPAR-associated transcriptional program in LGG. Transcriptome-wide Spearman correlation analyses were performed independently within WHO grade II (left panel) and WHO grade III (right panel) tumors. For each molecular subtype, an AMPAR expression score was calculated as the mean expression of *GRIA1*, *GRIA2*, *GRIA3*, and *GRIA4*, and genes were ranked according to their Spearman correlation with the subtype-specific AMPAR score. The 30 genes showing the strongest positive correlations are displayed together with their correlations with the individual AMPAR subunit genes.
